# Supplementary material for: Electronic cigarettes as a smoking cessation aid for patients with cancer: beliefs and behaviours of clinicians in the UK
Source: BMJ Open. 2020 Nov 19;10(11):e037637. doi: 10.1136/bmjopen-2020-037637 (PMC7678366; doi:10.1136/bmjopen-2020-037637)
Supplement: Author's manuscript [file bmjopen-2020-037637.draft_revisions.pdf]

BMJ Open is committed to open peer review. As part of this commitment we make the peer review history of every article we publish publicly available.

When an article is published we post the peer reviewers' comments and the authors' responses online. We also post the versions of the paper that were used during peer review. These are the versions that the peer review comments apply to.

The versions of the paper that follow are the versions that were submitted during the peer review process. They are not the versions of record or the final published versions. They should not be cited or distributed as the published version of this manuscript.

BMJ Open is an open access journal and the full, final, typeset and author-corrected version of record of the manuscript is available on our site with no access controls, subscription charges or pay-per-view fees (http://bmjopen.bmj.com).

If you have any questions on BMJ Open's open peer review process please email info.bmjopen@bmj.com

BMJ Open

Electronic cigarettes as a smoking cessation aid for cancer patients: beliefs and behaviours of health professionals

Journal:	*BMJ Open*
Manuscript ID	bmjopen-2020-037637
Article Type:	Original research
Date Submitted by the Author:	20-Feb-2020

Complete List of Authors:	Brett, Jo ; Oxford Brookes University, Health & Life Sciences<br>Davies, EL ; Oxford Brookes University,<br>Matley, Fiona; Oxford Brookes University, Health & Life Sciences<br>Aveyard, Paul; University of Oxford, Primary Care Health Sciences<br>Wells, Mary; Imperial College Healthcare NHS Trust, Faculty of Medicine, Department of Surgery & Cancer<br>Foxcroft, David; Oxford Brookes University UK, Psychology Department<br>Nicholson, Brian; University of Oxford, Nuffield Dept Primary Care Health Sciences<br>De Silva Minor, Shiroma; Oxford University Hospitals NHS Foundation Trust<br>Sinclair, Lesley; University of Glasgow<br>Jakes, Sarah; Vaper Representative<br>Watson, Eila; Oxford Brookes University, Clinical Health Care

Keywords:	EPIDEMIOLOGY, Health policy < HEALTH SERVICES ADMINISTRATION & MANAGEMENT, Quality in health care < HEALTH SERVICES ADMINISTRATION & MANAGEMENT, ONCOLOGY, PUBLIC HEALTH, MEDICAL EDUCATION & TRAINING

SCHOLARONE™
Manuscripts

BMJ

I, the Submitting Author has the right to grant and does grant on behalf of all authors of the Work (as defined in the below author licence), an exclusive licence and/or a non-exclusive licence for contributions from authors who are: i) UK Crown employees; ii) where BMJ has agreed a CC-BY licence shall apply, and/or iii) in accordance with the terms applicable for US Federal Government officers or employees acting as part of their official duties; on a worldwide, perpetual, irrevocable, royalty-free basis to BMJ Publishing Group Ltd ("BMJ") its licensees and where the relevant Journal is co-owned by BMJ to the co-owners of the Journal, to publish the Work in this journal and any other BMJ products and to exploit all rights, as set out in our licence.

The Submitting Author accepts and understands that any supply made under these terms is made by BMJ to the Submitting Author unless you are acting as an employee on behalf of your employer or a postgraduate student of an affiliated institution which is paying any applicable article publishing charge ("APC") for Open Access articles. Where the Submitting Author wishes to make the Work available on an Open Access basis (and intends to pay the relevant APC), the terms of reuse of such Open Access shall be governed by a Creative Commons licence – details of these licences and which Creative Commons licence will apply to this Work are set out in our licence referred to above.

Other than as permitted in any relevant BMJ Author's Self Archiving Policies, I confirm this Work has not been accepted for publication elsewhere, is not being considered for publication elsewhere and does not duplicate material already published. I confirm all authors consent to publication of this Work and authorise the granting of this licence.

For peer review only

Electronic cigarettes as a smoking cessation aid for cancer patients: beliefs and behaviours of health professionals

Brett J[1], Davies E[1], Matley F[1], Aveyard P[2], Wells M[3], Foxcroft D[1], Nicholson B[2], De Silva Minor S[4], Sinclair L[5], Jakes S[6], Watson E[1]

[1]Oxford Brookes University
[2]University of Oxford
[3]Imperial College Healthcare NHS Trust
[4]Oxford University Hospitals NHS Foundation Trust
[5]University of Edinburgh
[6] Vaper Representative

Corresponding author: Brett J. JBrett@brookes.ac.uk
Funder: Cancer Research UK

Dr Jo Brett	Senior Research Fellow, Supportive Cancer Care, Health and Life Sciences, Oxford Brookes University
Dr Emma Davies	Senior Lecturer in Psychology, Department of Psychology, Social Work and Public Health Oxford Brookes University
Fiona Matley	Research Fellow, Department of Psychology, Social Work and Public Health Oxford Brookes University
Prof. Paul Aveyard	Professor of Behavioural Medicine Nuffield Department of Primary Care Health Sciences, University of Oxford
Prof. Mary Wells	Lead Nurse for Research at Imperial College Healthcare NHS Trust and a Professor of Practice in Cancer Nursing at Imperial College, Faculty of Medicine, Department of Surgery & Cancer
Prof. David Foxcroft	Professor of Community Psychology and Public Health, Department of Psychology, Social Work and Public Health, Oxford Brookes University
Dr Brian Nicholson	Senior Clinical Researcher and Macmillan GP, Nuffield Department of Primary Care Health Sciences, University of Oxford
Dr Shiroma De Silva Minor	Consultant Clinical Oncologist, Oxford University Hospitals NHS Foundation Trust, Oxford
Lesley Sinclair	Research Fellow, Molecular, Genetic and Population Health Sciences, University of Edinburgh
Sarah Jakes	Vaper Representative
Prof Eila Watson	Prof of Supportive Cancer Care, Supportive Cancer Care, Health and Life Sciences, Oxford Brookes University

For peer review only - http://bmjopen.bmj.com/site/about/guidelines.xhtml

Abstract:

Objectives: To explore clinicians' beliefs and behaviours around recommending e-cigarettes as a smoking cessation aid for cancer patients

Design: Cross-sectional online survey

Setting: England, Wales, Scotland and Northern Ireland

Participants: Health professionals involved in the care of patients with cancer

Primary and secondary outcomes: Behavioural Change Wheel COM-B, knowledge, beliefs, current practice around e-cigarettes and other smoking cessation practices

Method: Clinicians (n=506) completed an online survey to assess beliefs and behaviours around e-cigarettes and other smoking cessation practices for cancer patients. Behavioural factors associated with recommending e-cigarettes in practice were assessed.

Results: 29% of clinicians would not recommend e cigarettes to cancer patients who continue to smoke. Factors associated with recommendation include smoking cessation knowledge (OR =0.64, CI 0.42-0.99) and e-cigarette knowledge (OR =1.64, CI 1.06-2.55), engagement with patients regarding smoking cessation (OR =2.12, CI 1.12-4.03), belief in the effectiveness of e-cigarettes (OR =2.36 CI 1.61-3.47), and belief in sufficient evidence on e-cigarettes (OR = 0.48, CI 0.25-0.91) and how comfortable they felt discussing e-cigarettes with patients (OR = 1.57 CI 1.04-2.36).

Conclusion: Many clinicians providing cancer care to patients who smoke do not recommend e-cigarettes as a smoking cessation aid and were unaware of national guidance supporting recommendation of e-cigarettes as a smoking cessation aid.

Strengths and limitations of this study

- This study reports an online survey with 506 clinicians to assess their beliefs and current practices around e-cigarettes as a smoking cessation aid for cancer patients.

For peer review only - http://bmjopen.bmj.com/site/about/guidelines.xhtml

- The study highlights that despite the popularity of e-cigarettes as a smoking cessation aid and support from Public Health England, clinicians have reservations about supporting their use in cancer patients
- Improved knowledge through training and change of policies at local and regional NHS sites are needed to encourage endorsement of the use of e-cigarettes in cancer patients
- Limitations include the sampling method and the potential for response bias

Key words: E cigarettes, cancer, smoking cessation, behavioural change, current practice

For peer review only

Background

Smoking is a well-established risk factor for many common cancers.[1-3] The adverse effects of smoking continue after a cancer diagnosis, increasing the risk of treatment-related complications, recurrence, the development of a second primary cancer, and mortality from both cancer-related and non–cancer-related causes.[4-11] Despite the increased risk of complications of cancer treatment, recurrence, and death, many patients with smoking-related cancers continue smoking following diagnosis[12-15] having tried and failed to stop smoking. Effective aids for cessation are available, but support to quit is not routinely offered as part of cancer care.[16,17] One study found that 39% (N=1129) of lung cancer patients, 37% (N=281) upper aero-digestive tract cancer patients, and 49% (N=850) of bladder cancer patients continued to smoke one year after diagnosis, figures that were likely to be higher as a third of the potential participants' smoking status was unknown.[18] To enhance the length and health-related quality of their lives, efforts are needed to support cancer patients to stop smoking cigarettes.

In recent years e-cigarettes have grown in popularity as a cessation aid among smokers worldwide. In the UK, e-cigarettes have swiftly become the most popular smoking cessation product for smokers.[19,20] There are 3.6 million EC users in the UK, of which only 0.8% have never smoked.[ASH 2019] E-cigarettes are used in 30% of quit attempts with currently around 20% of smokers and 30% of recent ex-smokers using them.[21] Evidence shows that e-cigarettes help smokers to stop smoking long-term[22-24] and recent evidence suggests that e-cigarettes are more effective for smoking cessation than nicotine-replacement therapy, when both products are accompanied by behavioural support.[25] Consequently, Public Health England, medical organisations and leading cancer charities in the UK recommend that clinicians support the use of e-cigarettes in patients who smoke.[26-29] Qualitative

For peer review only - http://bmjopen.bmj.com/site/about/guidelines.xhtml

evidence implies that clinicians may be more uncertain than guidance suggests is appropriate, with some expressing hostility and reporting practices that contradict the evidence.[30]

The aims of this study were to understand clinicians' beliefs and behaviours related to e-cigarettes for patients with cancer who continue to smoke, and to understand the behavioural factors that may promote or inhibit recommending e-cigarettes.

Methods

Design

The study was a cross-sectional online survey of health professional's knowledge, beliefs and current practice of smoking cessation and e-cigarettes. The survey was sent to clinicians involved in the cancer care pathway, working in primary and secondary care.

Survey development

The survey was developed using methods suggested by Bowling (1997).[31] The questions drew on 1) a literature review to identify evidence of clinicians' knowledge, attitudes, behaviours and current practice with respect to smoking cessation interventions, including e-cigarettes, in cancer patients; 2) expert opinion; 3) drawing on Behaviour Change Wheel (BCW).[32] Development also drew on previous survey questions exploring attitudes to e-cigarettes.[22,33] The questionnaire was piloted with five GPs and five cancer specialists, resulting in minor modification of wording of some questions.

The final questionnaire was structured according to the COM-B behaviour model based on Mitchie's Behaviour Change Wheel for development of interventions.[32] This model proposes that people need capability (C), opportunity (O) and motivation (M) to perform a behaviour (B) and was developed to guide understanding of behaviour in context and develop behavioural targets. The model proposes that for someone to engage in a particular

For peer review only - http://bmjopen.bmj.com/site/about/guidelines.xhtml

behaviour (B) at a given moment they must be physically able and psychological ability (C) and have the social and physical opportunity (O) to enact the behaviour and, in addition, want or need to enact the behaviour more than any other competing behaviours at that moment (M). This inclusive definition of motivation (M) covers basic drives and automatic processes such as habit and impulses as well as reflective processes including intention and choice. If a desired behaviour is not occurring (or an undesirable behaviour occurring) then an analysis of the determinants of the behaviour will help to define what needs to shift in order for the desired behaviour to occur (or the unwanted behaviour to cease).

The questionnaire included items relating to psychological capability (knowledge of smoking status of patients, knowledge and skills about smoking cessation in general, knowledge and skills about of e-cigarettes,); physical opportunity (time constraints talking to patients about e-cigarettes, physical constraints due to policy); social opportunity (relationship with patient and how this impacts on smoking cessation advice given, social norms e.g. most my colleagues support use of e-cigarettes, most my colleagues feel uncomfortable recommending e-cigarettes, clinicians should discourage smoking); reflective motivation (motivation to engage in smoking cessation with cancer patients, beliefs about how effective e-cigarettes are for cancer patients, beliefs about the HP's role, beliefs about the evidence base around e-cigarettes, attitudes towards e-cigarettes, beliefs about the harms of e-cigarettes particularly in comparison to tobacco cigarettes, and automatic motivation (how comfortable they feel giving smoking cessation advice in general and in giving specific advice on e-cigarettes to patients). A full copy of the questionnaire is available electronically (insert link here)

Sample:

For peer review only - http://bmjopen.bmj.com/site/about/guidelines.xhtml

MedeConnect, the survey arm of Doctors.net.uk asked clinicians who care for cancer patients in the UK (surgeons, oncologists, cancer nurse specialists, GPs and practice nurses) to complete the survey if they were on their existing databases and had consented to participate in research. The survey was completed between Nov 2018 and February 2019. A small financial reward was offered. Quota sampling was used to stratify the sample by type of clinician and by the seven National Health Service (NHS) UK regions. Participants confirmed they had read the participant information and consented to take part via email before the survey link was sent.

Analyses

Anonymised electronic responses were imported into SPSS (version 25) for analysis. Frequencies and proportions were used to summarise questionnaire responses. Results are reported using the COM-B model: physical and psychological capability; physical and social opportunity; and automatic and reflective motivation

Means and standard deviations for each measure for the whole sample were calculated and then compared by health professional occupation. Chi squared tests were used to compare the HPs on categorical measures. One way ANOVA was used to compare means for the other measures. Multiple comparisons were taken into account using a Bonferroni correction. Student-Newman-Keuls post-hoc tests were used to examine differences reported.

Binomial logistic regression models were used to predict the likelihood of recommending e-cigarettes to cancer patients. The dependent variable was dichotimised by always/nearly always /often recommend e-cigarettes (Q5_3) vs. sometimes/ infrequently/ never.

Ethics: This study was approved by the Oxford Brookes University Research Ethics Committee (2017 44 Brett)

For peer review only - http://bmjopen.bmj.com/site/about/guidelines.xhtml

Results:

Characteristics of respondents

The online survey was completed by 506 clinicians: 103 GPs, 102 oncologists, 100 cancer surgeons, 102 practice nurses and 99 cancer nurse specialists (CNSs). Table 1 describes the clinicians' characteristics:

Table 1 Clinician Characteristics

**Demographics**	All participants % (N = 506)	Primary Care % (n = 205)	Secondary Care % (n = 301)
Gender			
Male	41.1	35.1	45.2
Female	57.5	63.9	53.2
Prefer not to say	1.4	1.0	1.7
Mean year qualified as a health professional	1995 (1968-2009)	1994	1996
Role			
General practitioner (GP)	20.4	40.5	
Practice nurse	20.2		
Cancer surgeon	19.8		59.5
Oncologist	20.2		
Cancer nurse specialist	19.6		
NHS region			
London	15.8	12.2	18.3
South of England	21.1	21.5	20.9
Midlands & East SEA	25.5	25.4	25.6
North of England	20.6	22.9	18.9
Scotland	12.1	13.7	11.0

For peer review only

For peer review only - http://bmjopen.bmj.com/site/about/guidelines.xhtml

Wales	2.2	2.4	2.0
Northern Ireland	2.8	2.0	3.3
Main cancer group they care for			
All	37.0	76.1	10.3
Breast	24.1	12.2	32.2
Prostate	19.4	13.7	23.3
Lung / mesothelioma	18.0	11.7	22.3
Bowel	11.9	5.4	16.3
Kidney	11.9	0.5	19.6
Bladder	11.3	0.5	18.6
Other cancer groups	< 10	<5	<15
Smoking status of clinicians			
Smoke tobacco cigarettes	2.0	1.5	2.3
** Use e-cigarettes	1.2	1.5	1.0
Ex-smoker	21.3	22.9	20.3
Never smoker	72.3	72.2	72.4
Other	1.4	1.5	1.3
Prefer not to say	3.6	2.4	4.3

Reported behaviour: 29% (n=147) of clinicians would not recommend e-cigarettes to cancer patients who smoke. 51% (n=258) would recommend e-cigarettes as an interim measure, to help patients stop smoking completely, while 20% (n=101) would recommend e cigarettes as a partial replacement for smoking tobacco.

Psychological capability

Most clinicians (78%, n=394) knew the smoking status of their cancer patients, and routinely recorded their smoking status (73% n=368). 67% (n=339) reported that they routinely recommended patients stop smoking, or cut down (52%, n=263). 29% (n=147) referred

For peer review only

For peer review only - http://bmjopen.bmj.com/site/about/guidelines.xhtml

patients to the NHS stop smoking services, 14% (n=71) recommended NRT, 9% (n=46) recommended digital smoking cessation tools, and 5% (n=25) prescribed medication (Varenicline or Bupropion).

Many clinicians felt they had insufficient knowledge (57%, n=286) and training (73%, n=370) to provide advice about e-cigarettes to patients and a further 36% (n=182) indicated that they did not know the efficacy of e-cigarettes with regard to smoking cessation.

Clinicians derived information about e-cigarettes from several sources. Overall, only 9.5% of health professionals knew whether their organisation had guidance concerning advice to patients on e-cigarette use. Most health professionals had sought information about e-cigarettes from government/health agencies (55%), but also from professional associations (37%), healthcare colleagues (29%), news/media/advertising (24%), scientific literature (23%), professional development/training (22%) and charities (18%). Nineteen percent of health professionals had never sought information about e-cigarettes. One quarter of respondents (25%, n=124) were uncertain whether e-cigarettes were less harmful than smoking tobacco, while 10% (n=52) thought e-cigarettes were equally harmful or more harmful than smoking tobacco. 18% (n=93) considered using e-cigarettes to be more harmful than regular nicotine replacement therapies (e.g. gum, nasal spray, patches) and 54% (n=273) were uncertain.

Physical opportunity

The majority of health professionals (56%, n=285) said that e-cigarette use was prohibited in all areas at their main place of work. Twenty three percent (n=119) reported that the use of e-cigarettes was permitted, with 24% (n=29) reporting use in designated smoking areas only. Overall 51% (n=258) agreed that time constrained their ability to talk about e-cigarettes with patients.

For peer review only - http://bmjopen.bmj.com/site/about/guidelines.xhtml

Social opportunity

The nature of the relationships between health professionals and patients with cancer were important in whether and how smoking cessation was discussed. 55% (n=278) of health professionals reported that having a good relationship would make them more likely to speak to their patient about stopping or cutting down, while 45% (n=228) reported that having a poor relationship would make them less likely to discuss smoking cessation. Many clinicians (42% n= 212) felt uncomfortable when asked by patients for an opinion on e-cigarettes. The large majority, 82%, had been asked about e-cigarettes by patients in the past year (2017/18), up from 21% in 2016/17.

Thirty eight percent (n=192) said that most of their colleagues would feel uncomfortable recommending e-cigarettes to patients with cancer, and 37% (n=187) were unsure whether health professionals should discourage patients with cancer from using e-cigarettes.

Automatic motivation

Subconscious biases towards e-cigarettes were influenced by health professionals' beliefs around the effectiveness of and evidence on e-cigarettes, as reported in the psychological capability section above.

Clinicians reported that their decisions to speak with patients with cancer about smoking cessation were influenced by their perceptions of the patient. Health professionals reported that they were more likely to discuss smoking cessation if they judged the person was motivated to quit (69%, n=349), or was coping well (67%, n=339).

Reflective Motivation

Reflective motivation related to the clinicians' perceived role in smoking cessation and national and organisational policy on e-cigarettes. Two thirds (65%, n=327) of clinicians agreed that they should play a greater role in helping cancer patients stop smoking. Health

For peer review only - http://bmjopen.bmj.com/site/about/guidelines.xhtml

professionals' were divided over whether e-cigarettes should be licensed and available on prescription for patients with cancer, with 39% (n=199) respondents disagreeing, and 32% (n=162) saying they should be available on prescription. Furthermore, 30% (n=150) of clinicians felt that public health campaigns, such as Stoptober should not endorse using e-cigarettes as a way to give up smoking tobacco, while 29% (n=149) thought e-cigarettes should be endorsed in campaigns.

Differences in e-cigarettes practice between health professionals

GPs and practice nurses were significantly more likely to say that they recommended e-cigarettes to cancer patients than the other HPs included in the study (see Table 2). GPs and practice nurses also rated themselves as having significantly greater general knowledge and skills than other professionals to help patients with cancer stop smoking, and reported that they had more time to discuss smoking with patients and having an important role in helping patients cut down.

Practice nurses engaged in significantly more behaviours (e.g. knew smoking status, engaged in advice to patients, supported e-cigarette use in patients) related to smoking cessation with patients than all the other groups. GPs engaged in significantly more behaviours than the other HPs, but fewer than practice nurses.

Practice nurses were significantly more likely to believe in the effectiveness of e-cigarettes in helping cancer patients stop smoking compared to other HPs.

Table 2: **COM-B measures by HP– Mean and SD**

	Total	General Practitioner	Practice Nurse	Cancer Surgeon	Oncologist	Cancer Nurse Specialist	Test statistic, p
N of participants	506	103	102	100	102	99	

For peer review only - http://bmjopen.bmj.com/site/about/guidelines.xhtml

Recommend e-cigarettes always/often, N (%)	97 (19.2)	24 (23.3%)	36 (35.3)	9 (9)	16 (15.7)	12 (12.1)	χ^2 = 28.90, p<.001
**Capability**							
Know status	4.72 (.59)	4.75 (.48)	4.78 (.46)	4.81 (.46)	4.75 (.60)	4.52 (.81)	F = 4.17, p=.002*
General smoking knowledge	3.40 (1.03)	3.80 (.86)	3.75 (1.08)	3.10 (1.00)	3.17 (1.03)	3.19 (.95)	F = 12.09, p<.001*
E-cig knowledge	2.34 (.99)	2.40 (.89)	2.45 (1.07)	2.21 (.94)	2.54 (1.05)	2.11 (.95)	F = 3.32, p=.011
**Opportunity**							
Time	2.60 (1.03)	2.46 (.95)	2.87 (.94)	2.39 (1.00)	2.29 (1.06)	3.02 (1.00)	F= 10.47, p<.001*
Patient relationships	2.51 (.68)	2.45 (.72)	2.52 (.66)	2.59 (.64)	2.49 (.67)	2.52 (.72)	F = .59, p=.668
Social norms	3.00 (.72)	2.98 (.86)	3.18 (.75)	2.85 (.66)	3.08 (.65)	2.91 (.64)	F =3.36, p=.010
**Motivation**							
Engagement with patients who smoke	2.77 (.61)	2.90 (.55)	3.21 (.55)	2.60 (.54)	2.55 (.58)	2.59 (.55)	F= 26.13, p<.001*
Effectiveness in helping cancer pts	2.83 (1.50)	2.86 (1.48)	3.39 (1.43)	2.49 (1.54)	2.81 (1.45)	2.57 (1.44)	F = 5.89, p<.001*
Importance of HP role	4.35 (.76)	4.48 (0.62)	4.60 (.60)	4.33 (.83)	4.20 (.81)	4.15 (.84)	F =6.40, p<.001*
Lack of evidence N (%)	242 (47.8)	57 (55.3)	51 (50)	44 (44)	41 (40.2)	50 (50.5)	χ^2 =5.60 p=.231
Attitudes	3.22 (.61)	3.23 (.64)	3.39 (.62)	3.08 (.61)	3.26 (.53)	3.13 (.59)	F =.4.04, p=.003
Harm	3.16 (.68)	3.25 (.63)	3.15 (.67)	3.18 (.66)	3.22 (.71)	3.01 (.71)	F =1.32, p=.263
Better than smoking	3.86 (.96)	4.00 (1.00)	3.84 (1.00)	3.82 (.81)	4.07 (.86)	3.56 (1.05)	F = 4.39, p=.002*
Comfortable discussing	4.40 (.76)	4.45 (.75)	4.44 (.71)	4.49 (.72)	4.39 (.81)	4.24 (.80)	F =1.59, p=.175

For peer review only

For peer review only - http://bmjopen.bmj.com/site/about/guidelines.xhtml

smoking in general							
Comfortable discussing e-cigs	2.91 (1.16)	3.09 (1.13)	3.04 (1.23)	2.65 (1.17)	3.00 (1.11)	2.76 (1.12)	F =2.79, p=.026

Note * = significant for F tests when multiple comparisons taken into consideration, accepted p value corrected to 0.003

Logistic regression model

Table 3 presents a logistic regression model including all COM-B factors and controlling for health professional type. In this model, capability factors of smoking knowledge (OR =0.64 CI 0.42-0.99) and e-cigarette knowledge (OR =1.64 CI 1.06-2.55) significantly predicted recommending e-cigarettes to cancer patients. Specifically, those with lower levels of knowledge and skills about smoking cessation in general were less likely to recommend e-cigarettes, and those with higher levels of knowledge and skills about e-cigarettes were more likely to recommend them. Motivation factors were also important - engagement with patients regarding smoking (OR =2.12 CI 1.12-4.03), belief in the effectiveness of e-cigarettes (OR =2.36 CI 1.61-3.47), belief in sufficient evidence on e-cigarettes (OR = 0.48 CI 0.25-0.91) and the social opportunity factor of how comfortable they felt discussing e-cigarettes with patients (OR = 1.57 CI 1.04-2.36) all significantly predicted recommending e-cigarettes to cancer patients. Specifically, those who reported higher levels of engagement around smoking cessation, and those who were comfortable discussing e-cigarettes were more likely to recommend them. However, those who felt the evidence base was lacking were less likely to recommend them.

Table 3: Results of full binary logistic regression model exploring all factors relating to capability, opportunity and motivation to recommend e-cigarettes to cancer patients

				95% CI for Odds Ratio		
	B	Wald(df =1)	p	Lower	Odds Ratio	Upper
**Capability**						
Know status	0.758	3.32	0.068	0.944	2.134	4.821

For peer review only - http://bmjopen.bmj.com/site/about/guidelines.xhtml

General smoking knowledge	-0.44	3.866	0.049	0.416	0.644	0.999
E-cig knowledge	0.495	4.878	0.027	1.057	1.641	2.546
**Opportunity**						
Time	0.083	0.237	0.626	0.778	1.087	1.517
Patient relationships	-0.092	0.128	0.72	0.551	0.912	1.51
Social norms	-0.047	0.028	0.867	0.549	0.954	1.658
**Motivation**						
Engagement with patients who smoke	0.752	5.308	0.021	1.119	2.122	4.025
Effectiveness in helping cancer pts	0.858	19.09	0.000	1.605	2.359	3.466
Importance of HP role	0.259	0.834	0.361	0.743	1.295	2.257
Sufficient evidence	-0.745	4.963	0.026	0.247	0.475	0.914
Attitudes	0.37	1.038	0.308	0.711	1.447	2.947
Harm	0.023	0.006	0.941	0.556	1.023	1.885
Better than smoking	-0.009	0.001	0.972	0.593	0.991	1.655
Comfortable discussing smoking in general	-0.402	2.18	0.14	0.392	0.669	1.141
Comfortable discussing e-cigs	0.448	4.594	0.032	1.039	1.565	2.356
**Health professional type**						
		5.385	0.25			
GP	0.276	0.256	0.613	0.453	1.318	3.839
Practice Nurse	0.281	0.294	0.588	0.479	1.324	3.659
Cancer Surgeon	-0.805	1.791	0.181	0.138	0.447	1.453
Oncologist	-0.391	0.514	0.473	0.232	0.676	1.971
**Constant**	-11.416	20.331	0		0	

Note: reference categories - for HP type = Cancer Nurse Specialist, for sufficient evidence = yes

Discussion

The findings from this study suggest relatively low levels of clinician support around recommending e-cigarettes to cancer patients. Despite a growing evidence base to support use and popularity of e-cigarettes as a smoking cessation aid in the UK nearly a third of clinicians do not recommend e-cigarettes to cancer patients who smoke. Not recommending e-cigarettes is associated with a lack of knowledge regarding smoking cessation and e-cigarettes, lack of engagement in smoking cessation practices with patients that smoke, low belief in effectiveness of e-cigarettes, low belief in evidence around e-cigarettes, and not feeling comfortable discussing e-cigarettes with their patients.

For peer review only

For peer review only - http://bmjopen.bmj.com/site/about/guidelines.xhtml

In line with the results of this study, a survey of members of The British Thoracic Oncology Group in April 2015 showed that the vast majority of clinicians agreed that they needed more information and guidance regarding e-cigarettes to advise patients (n = 124, 92.6 %). Clinicians lacked confidence in providing advice to patients with lung cancer over the use of e-cigarettes.[33] Our findings indicate that three years on clinicians continue to lack knowledge and confidence in recommending e-cigarettes to cancer patients despite a public health policy to support e-cigarettes. Clinicians across disease groups have reported a clear need for training and local guidance around e-cigarettes in line with the national public health policy.[35-37] Interventions are needed to target the reported behavioural factors associated with clinicians' recommendation of e-cigarettes.

This study highlights that the COM-B components of psychological capability, social opportunity and motivation were all associated with clinicians' beliefs and behaviours around recommending e-cigarettes. Improving knowledge through accessible training on e-cigarettes alongside local adoption of public health policies around e-cigarettes throughout the NHS may support clinicians to feel more confident and comfortable in recommending them to patients. NICE advise that health professionals have an informed discussion with patients on use of e-cigarettes to stop smoking.[29]

Opportunity to recommend e-cigarettes could be improved by providing a more positive strategy around e-cigarettes. An ongoing ban of e-cigarettes in many NHS organisations, or permitted use only in dedicated smoking areas not only limits clinicians from demonstrating e-cigarettes, but puts vapers at risk of relapsing. Allowing vaping in appropriate parts of the NHS may improve compliance with public health policy around e-cigarettes.

For peer review only - http://bmjopen.bmj.com/site/about/guidelines.xhtml

A recent study reports that practitioners suggested the development of decision aids around e-cigarettes, such as a leaflet, booklet or online resource to use during consultations with patients.[38] This would aid a more 'neutral' decision around use of e-cigarettes and potentially improve confidence around discussions on e-cigarettes.[30] Furthermore, engagement from local Clinical Commissioning Groups (CCGs) and clinical leads alongside accessible training is needed to encourage clinicians to support use of e-cigarettes in cancer patients.

To date there is still no medicinally licenced EC in the UK, or anywhere else in the world, which is possibly presenting challenges for health professionals wishing to demonstrate e-cigarettes or recommend for inpatient use. In this study, clinicians were divided over this decision, with two fifths of clinicians disagreeing, and nearly a third agreeing to licensing of e-cigarettes for medicinal uses. Health professionals may be more comfortable recommending e-cigarettes if they were available on prescription.

Debate is also needed on how e-cigarettes could be integrated into smoking cessation practices of cancer clinicians for patients with cancer. Smoking cessation practices are already well developed in other disease groups such as coronary heart disease (CHD).[39] It is therefore timely to set out the role of a smoking cessation service within the cancer pathway, including the role of e-cigarettes can play in helping cancer patients to quit smoking long term after a diagnosis.[22] While attendance at a cancer clinic provides a prime opportunity for clinicians to provide smoking cessation advice to those who smoke, this is not currently routinely offered. Patients have highlighted the need for more specific stop smoking cessation advice for those diagnosed with cancer, and have reported difficulty in attending external smoking cessations services in addition to all their other clinic appointments.[12] Smoking cessation advice has historically been the role of primary care or

For peer review only - http://bmjopen.bmj.com/site/about/guidelines.xhtml

community smoking cessation services, and this is reflected in the greater knowledge, confidence and more positive attitude towards e-cigarettes among practice nurses and GPs reported in this study. However, lack of funding has seen a decline in these services, and alternatives are needed. Maintaining a trusting and caring relationship with their patient may discourage clinicians from sensitive discussions around quitting smoking[40], although clinicians misconceptions of patients abilities to stop smoking have also been reported,[41] and patients may stop smoking in the short term during treatment, but return to smoking post treatment.

Strengths and limitations

To our knowledge, this study is the first to examine a broad range of clinicians' behaviours and beliefs around the use of e-cigarettes in patients across different cancer groups. The COM-B model has previously been successfully used in the development of smoking cessation interventions.[42-44] In this study, it has enabled the identification of factors, which could be used to improve clinicians' recommendation of e-cigarettes.

The recruitment procedure for this survey utilised an existing network of electronically active clinicians from the research arm of doctors.net.com. This method has the advantage of speed and guaranteed response which is beneficial considering that surveys with busy clinicians have commonly suffered from poor response rates. However, this sample may not be representative of the population of clinicians in the United Kingdom. Quota sampling ensured diversity in terms of geographical spread, gender and years of clinical experience, although our sample included clinicians who worked with patients who had a wide range of cancer diagnoses, and not just those with cancers directly associated with smoking

The potential for response bias should be considered; for example, those with a greater interest in smoking cessation for cancer patients may have been more likely to respond,

For peer review only - http://bmjopen.bmj.com/site/about/guidelines.xhtml

For peer review only

whereas clinicians who are smokers may have been under-represented. In addition, findings rely on self-report.

Conclusions

Despite the evidence that e-cigarettes help smokers quit smoking, and the positive public health stance towards e-cigarettes in the UK, clinicians remain cautious about recommending e-cigarettes to cancer survivors who continue to smoke. Improved knowledge through training and change of policies at local and regional NHS sites are needed to encourage endorsement of the use of e-cigarettes in cancer patients.

For peer review only

For peer review only - http://bmjopen.bmj.com/site/about/guidelines.xhtml

Acknowledgements

This work was funded and supported by Cancer Research UK. We thank the clinicians who participated in this online survey, and Medeconnect Healthcare Insight, the research arm of Doctors.Net.UK for recruiting, sending and collating results for the online survey.

Summary results have been presented at National Cancer Research Institute (NCRI) conference Glasgow Nov 2018, and International Psycho-Oncology Society (IPOS) conference in Banff, Canada, Sept 2019

E-cigarettes as a Smoking Cessation aid in Cancer Patients: Health Professionals knowledge, attitude and current practice. NCRI 2018 abstract 2171

E-cigarettes as a Smoking Cessation aid in Cancer Patients: Health Professionals knowledge, attitude and current practice. IPOS 2019 abstract 751. Journal of Psychosocial Oncology – Research and Practice Sept 2019, vol 1: suppl 1 1S

Author Contributions

JB and EW designed the study. All authors were involved in the development of the protocol. ED and FM conducted the statistical analysis. All authors were involved in the interpretation of the data and edited the manuscript.

Competing interests: The authors have no conflict of interest or disclosures. All authors declare no financial or other relationships or activities that could appear to have influenced the submitted work.

Ethical approval:

This study complies with ethical standards. Informed consent was gained. The study obtained ethics approval to conduct the study from Oxford Brookes University Ethics

For peer review only - http://bmjopen.bmj.com/site/about/guidelines.xhtml

Committee (2017 44 Brett) and was performed in accordance with the Declaration of Helsinki.

Patient and Public Involvement statement: Cancer service users and vapor representatives were involved in the proposal development, questionnaire development and dissemination of the results of this study. One vaper representative was involved in the write up and is an author on the paper.

Funder:

Cancer Research UK Ref: A24355 Dr Jo Brett

Data sharing/Data availability:

Data will be deposited in the university repository, where request for access can be submitted. Data access can be requested from the corresponding author. The data will be kept for a minimum of ten years following publication.

For peer review only

References

1. Ordenez-Mena JM, Schottker B, on behalf of the Consortium on Health and Ageing: Network of Cohorts in Europe and the United States (CHANCES). Quantification of the smoking-associated cancer risk with rate advancement periods: meta-analysis of individual participant data from cohorts of the CHANCES consortium *BMC Medicine 2016*, 14 (62)
2. US Health and Human Resources. The Health Consequences of Smoking—50 Years of Progress: A Report of the Surgeon General 2016
3. US Health and Human Resources How Tobacco Smoke Causes Disease: The Biology and Behavioral Basis for Smoking-Attributable Disease A Report of the Surgeon GeneralAtlanta (GA): Centers for Disease Control and Prevention (US); 2010.
4. Yang BM Jacobs EJ, Gapstur SM, Stevens V, Cambell PT. Active Smoking and Mortality Among Colorectal Cancer Survivors: The Cancer Prevention Study II Nutrition Cohort. *J Clin Oncol* 2015, 33, 10.1200/JCO.2014.58.3831
5. Warren GW, Kasza KA, Reid ME, Cummings KM, Marshall JR. Smoking at diagnosis and survival in cancer patients. *Int J Cancer* 2013, 132,401–410.
6. Gajdos C, Hawn MT, Campagna EJ, Henderson WG, Singh JA, Houston T. Adverse effects of smoking on postoperative outcomes in cancer patients. *Ann Surg Oncol* 2012, 19,1430–1438
7. Gillison ML, Zhang Q, Jordan R, Xiao W, Westra WH, Trotti A, Spencer S, Harris J, Chung CH, Ang K. Tobacco smoking and increased risk of death and progression for patients with p16-positive and p16-negative oropharyngeal cancer. *J Clin Oncol* 2012, 30,2102–2111
8. Kenfield SA, Stampfer MJ, Chan JM, Giovannucci E. Smoking and prostate cancer survival and recurrence. *JAMA* 2011 305,2548–2555
9. Parsons A, Daley A, Begh R, Aveyard P. Influence of smoking cessation after diagnosis of early stage lung cancer on prognosis: systematic review of observational studies with meta-analysis. *BMJ* 2010, 340-348
10. Bittner N, Merrick GS, Galbreath RW, Butler WM, Wallner KE, Allen ZA, Brammer SG, Moyad M. Primary causes of death after permanent prostate brachytherapy. *Int J Radiat Oncol Biol Phys* 2008, 7,:433–440

For peer review only - http://bmjopen.bmj.com/site/about/guidelines.xhtml

11. Hooning MJ, Botma A, Aleman BM, Baaijens MH, Bartelink H, Klijn JG, Taylor CW, van Leeuwen FE. Long-term risk of cardiovascular disease in 10-year survivors of breast cancer. *J Natl Cancer Inst* 2007, 99,365–375
12. Wells M, Aitchison P, Harris F, Ozakinci G, Radley A, Bauld, L.. Williams, B.. Barriers and facilitators to smoking cessation in a cancer context: A qualitative study of patient, family and professional views. *BMC Cancer* 2017, 17(1), 348. doi: 10.1186/s12885-017-3344-z
13. Warren GW, Marshall JR, Cummings KM, Toll B, Gritz ER, Hutson A, Dibai S, Herbst R, Dresler C IASLC Tobacco Control and Smoking Cessation Committtee. Practice patterns and perceptions of thoracic oncology providers on tobacco use and cessation in cancer patients. *J Thorac Oncol* 2013,8(5),543–548
14. Park ER, Japuntich S, Temel J, et al. A smoking cessation intervention for thoracic surgery and oncology clinics: a pilot trial. *J Thorac Oncol* 2011, 6,1059–1065
15. Cooley M E, Sarna L., Kotlerman ., Lukanich JM, Jaklitsch M, Green S.B, Bueno R. Smoking cessation is challenging even for patients recovering from lung cancer surgery with curative intent. *Lung Cancer* 2009, 66(2), 218-225
16. Warren GW, Marshall JR, Cummings KM, Zevon MA, Reed R, Hysert P, Mahoney MC, et al. Automated tobacco assessment and cessation support for cancer patients. *Cancer* 2014,120(4),562–569
17. Murray R, Leonardi-Bee J, Marsh J, Jayes L, Britton J. Smoking status ascertainment and interventions in acute medical patients. *Clin Med* 2012,12,59-62.

18. Koshiaris C, Aveyard P, Oke J, Ryan R, Szatkowski L, Stevens R, Farley A.Smoking cessation and survival in lung, upper aero-digestive tract and bladder cancer: cohort study. *British Journal of Cancer* 2017, 117(8),1224-1232
19. Health and Social Care Information Centre (HSCIC) (2019). Statistics on NHS Stop Smoking Services in England - April 2018 to March 2019 Aug, 2019
20. Action on Smoking and Health (ASH). Use of e-cigarettes (vaporisers) among adults in Great Britain September 2019 https://ash.org.uk/wp-content/uploads/2019/09/Use-of-e-cigarettes-among-adults-2019.pdf Accessed Nov 2019

For peer review only - http://bmjopen.bmj.com/site/about/guidelines.xhtml

21. NHS Smokefree report: https://www.nhs.uk/smokefree/help-and-advice/e-cigarettes Dec 2018. Accessed May 2019

22. McNeill A, Brose LS, Calder R, Bauld L & Robson D. Evidence review of e-cigarettes and heated tobacco products 2018. A report commissioned by Public Health England. London: Public Health England 2018

23. McRobbie H, Bullen C, Hartmann-Boyce J, Hajek P. Electronic cigarettes for smoking cessation and reduction. *Cochrane Database Syst Rev* 2014, (12), Cd010216. doi: 10.1002/14651858.CD010216.pub2

24. Hajek P, Etter, JF, Benowitz N, Eissenberg T, McRobbie H. Electronic cigarettes: review of use, content, safety, effects on smokers and potential for harm and benefit. *Addiction* 2014, 109(11), 1801-1810. doi: 10.1111/add.12659

25. Hajek P, Phillips-Waller A, Przulj D, Myers Smith K, Bisal N, Li J, Parrott S, Sasieni P, Dawkins L, Ross L, Wu Q, McRobbie H J. A Randomized Trial of E-Cigarettes versus Nicotine-Replacement Therapy *N Engl J Med* 2019, 380,629-637

26. Royal College of Physicians (RCP) (2016). Nicotine without smoke: Tobacco harm reduction https://www.rcplondon.ac.uk/projects/outputs/nicotine-without-smoke-tobacco-harm-reduction. Accessed May 2019

27. Public Health England (McNeill A, Brose LS, Calder R, Hitchman SC, Hajek P, McRobbie H) (2014).

28. Cancer Research UK Policy Briefing: E Cigarettes. 2016 https://www.cancerresearchuk.org/sites/default/files/cruk_e-cigarette_briefing.pdf

29. NICE (2014). Tobacco: harm-reduction approaches to smoking. NICE public health guidance 45. http://guidance.nice.org.uk/PH45

30. Stepney M, Aveyard P, Begh R. GPs' and nurses' perceptions of electronic cigarettes in England: a qualitative interview study. *British Journal of General Practice* 2019, 69 (678), e8-e14.

31. Bowling A. Constructing and evaluating questionnaires for health services research. In: Research methods in health: investigating health and health services. Buckingham: Open University Press, 1997.

32. Michie S, van Stralen MM, West, R. (2011). The behaviour change wheel: A new method for characterising and designing behaviour change interventions. *Implementation Science* 2011, 6.doi: 10.1186/1748-5908-6-42

For peer review only - http://bmjopen.bmj.com/site/about/guidelines.xhtml

33. Sherratt FC, Newson L, Field JK. Electronic cigarettes: a survey of perceived patient use and attitudes among members of the British thoracic oncology group. *Respir Res* 2016;17:55.

34. Michie S, Atkins L, West R. The Behaviour Change Wheel: A Guide to Designing Interventions. London: Silverback Publishing 2014. www.behaviourchangewheel.com

35. Luxton NA, Hih P, Rahman MA. Electronic Cigarettes and Smoking Cessation in the Perioperative Period of Cardiothoracic Surgery: Views of Australian Clinicians *International Journal of Environmental Research and Public Health* 2018 15(11):248110.3390/ijerph15112481

36. Kanchastamsham V, Saladi S, Rodrigues J, Fernandes H, Patolia S, Santosh S. The knowledge, concerns and healthcare practices among physicians regarding electronic cigarettes. *J Community Hosp Intern Med Perspect*, 2017 Jul, 7(3), 144–150

37. Cummins S, Lieschow S, Bailey L, Bush T, Wassam K, Copeland L, Zhu SH. Knowledge and Beliefs about E cigarettes among Quitline Cessation Staff. *Addictive Behaviors*, 2016, 60,28-83

38. Stepney M, Aveyard P, Begh R. GPs and Nurses' perceptions of e-cigarettes in England. A Qualitative Study. *British Journal of General Practice* 2019, DOI: https://doi.org/10.3399/bjgp18X699821

39. Farley A, Koshiaris C, Oke J, Ryan R, Szatkowski L, Stevens R, Aveyard P. Physician Support of Smoking Cessation After Diagnosis of Lung, Bladder, or Upper Aerodigestive Tract Cancer. *Annals of family medicine* 2017 15(5), 443-450. doi: 10.1370/afm.2100

40. Butler CC, Pill R, Stott NCH. Qualitative study of patients' perceptions of doctors' advice to quit smoking: implications for opportunistic health promotion. *BMJ* 1998, 8,1878–1881

41. Sheals K, Tombor I, McNeill A, Shahab L. A mixed method systematic review and meta analysis of mental health professionals' attitiudes towards smoking and smoking cessation among people with mental illnesses. Addiction 2016,111,1536-53

42. Gould GS, Bar-Zeev Y, Bovill M, Atkins L, Grupetta M, Clarke MJ, Bonevski B. Designing an implementation intervention with the Behaviour Change Wheel for

For peer review only - http://bmjopen.bmj.com/site/about/guidelines.xhtml

health provider smoking cessation care for Australian Indigenous pregnant women. *Implementation Science* 2017, 12,114

43. Beenstock J, Sniehotta FF, White M, Bell R, Milne EM, Araujo-Soares V.. What helps and hinders midwives in engaging with pregnant women about stopping smoking? A cross-sectional survey of perceived implementation difficulties among midwives in the North East of England. *Implementation science* 2012, doi:10.1186/1748-5908-7-36

44. Wadsworth E, Neale J, McNeill A, Hitchman SC. How and Why Do Smokers Start Using E-Cigarettes? Qualitative Study of Vapers in London, UK. *Int J Environ Res Public Health* 2016 Jun 30,13(7) doi: 10.3390/ijerph13070661.

For peer review only

For peer review only - http://bmjopen.bmj.com/site/about/guidelines.xhtml

STROBE Statement—Checklist of items that should be included in reports of ***cross-sectional studies***

	Item No	Recommendation	Page No
**Title and abstract**	1	(*a*) Indicate the study's design with a commonly used term in the title or the abstract	2
		(*b*) Provide in the abstract an informative and balanced summary of what was done and what was found	2
**Introduction**			
Background/rationale	2	Explain the scientific background and rationale for the investigation being reported	4
Objectives	3	State specific objectives, including any prespecified hypotheses	5
**Methods**			
Study design	4	Present key elements of study design early in the paper	5
Setting	5	Describe the setting, locations, and relevant dates, including periods of recruitment, exposure, follow-up, and data collection	6,7
Participants	6	(*a*) Give the eligibility criteria, and the sources and methods of selection of participants	6,7
Variables	7	Clearly define all outcomes, exposures, predictors, potential confounders, and effect modifiers. Give diagnostic criteria, if applicable	5,6
Data sources/ measurement	8*	For each variable of interest, give sources of data and details of methods of assessment (measurement). Describe comparability of assessment methods if there is more than one group	5
Bias	9	Describe any efforts to address potential sources of bias	5,6
Study size	10	Explain how the study size was arrived at	
Quantitative variables	11	Explain how quantitative variables were handled in the analyses. If applicable, describe which groupings were chosen and why	7
Statistical methods	12	(*a*) Describe all statistical methods, including those used to control for confounding	7
		(*b*) Describe any methods used to examine subgroups and interactions	7
		(*c*) Explain how missing data were addressed	N/A
		(*d*) If applicable, describe analytical methods taking account of sampling strategy	N/A
		(*e*) Describe any sensitivity analyses	N/A
**Results**			
Participants	13*	(a) Report numbers of individuals at each stage of study—eg numbers potentially eligible, examined for eligibility, confirmed eligible, included in the study, completing follow-up, and analysed	8
		(b) Give reasons for non-participation at each stage	N/A
		(c) Consider use of a flow diagram	N/A
Descriptive data	14*	(a) Give characteristics of study participants (eg demographic, clinical, social) and information on exposures and potential confounders	8 Table1
		(b) Indicate number of participants with missing data for each variable of interest	N/A
Outcome data	15*	Report numbers of outcome events or summary measures	8,9,10,11
Main results	16	(*a*) Give unadjusted estimates and, if applicable, confounder-adjusted estimates and their precision (eg, 95% confidence interval). Make	8,9,10,11

For peer review only - http://bmjopen.bmj.com/site/about/guidelines.xhtml

		clear which confounders were adjusted for and why they were included N/A	
		(*b*) Report category boundaries when continuous variables were categorized	N/A
		(*c*) If relevant, consider translating estimates of relative risk into absolute risk for a meaningful time period	N/A
Other analyses	17	Report other analyses done—eg analyses of subgroups and interactions, and sensitivity analyses	10,11
**Discussion**			
Key results	18	Summarise key results with reference to study objectives	12
Limitations	19	Discuss limitations of the study, taking into account sources of potential bias or imprecision. Discuss both direction and magnitude of any potential bias	14,15
Interpretation	20	Give a cautious overall interpretation of results considering objectives, limitations, multiplicity of analyses, results from similar studies, and other relevant evidence	12-14
Generalisability	21	Discuss the generalisability (external validity) of the study results	14
**Other information**			
Funding	22	Give the source of funding and the role of the funders for the present study and, if applicable, for the original study on which the present article is based	18

*Give information separately for exposed and unexposed groups.

Note: An Explanation and Elaboration article discusses each checklist item and gives methodological background and published examples of transparent reporting. The STROBE checklist is best used in conjunction with this article (freely available on the Web sites of PLoS Medicine at http://www.plosmedicine.org/, Annals of Internal Medicine at http://www.annals.org/, and Epidemiology at http://www.epidem.com/). Information on the STROBE Initiative is available at www.strobe-statement.org.

For peer review only

For peer review only - http://bmjopen.bmj.com/site/about/guidelines.xhtml

BMJ Open

Electronic cigarettes as a smoking cessation aid for cancer patients: beliefs and behaviours of clinicians in the UK

Journal:	*BMJ Open*
Manuscript ID	bmjopen-2020-037637.R1
Article Type:	Original research
Date Submitted by the Author:	04-Sep-2020
Complete List of Authors:	Brett, Jo ; Oxford Brookes University, Health & Life Sciences<br>Davies, EL ; Oxford Brookes University,<br>Matley, Fiona; Oxford Brookes University, Health & Life Sciences<br>Aveyard, Paul; University of Oxford, Primary Care Health Sciences<br>Wells, Mary; Imperial College Healthcare NHS Trust, Faculty of Medicine, Department of Surgery & Cancer<br>Foxcroft, David; Oxford Brookes University UK, Psychology Department<br>Nicholson, Brian; University of Oxford, Nuffield Dept Primary Care Health Sciences<br>De Silva Minor, Shiroma; Oxford University Hospitals NHS Foundation Trust<br>Sinclair, Lesley; University of Glasgow<br>Jakes, Sarah; Vaper Representative<br>Watson, Eila; Oxford Brookes University, Clinical Health Care
**Primary Subject Heading**:	Oncology
Secondary Subject Heading:	Health services research, Public health
Keywords:	EPIDEMIOLOGY, Health policy < HEALTH SERVICES ADMINISTRATION & MANAGEMENT, Quality in health care < HEALTH SERVICES ADMINISTRATION & MANAGEMENT, ONCOLOGY, PUBLIC HEALTH, MEDICAL EDUCATION & TRAINING

SCHOLARONE™
Manuscripts

I, the Submitting Author has the right to grant and does grant on behalf of all authors of the Work (as defined in the below author licence), an exclusive licence and/or a non-exclusive licence for contributions from authors who are: i) UK Crown employees; ii) where BMJ has agreed a CC-BY licence shall apply, and/or iii) in accordance with the terms applicable for US Federal Government officers or employees acting as part of their official duties; on a worldwide, perpetual, irrevocable, royalty-free basis to BMJ Publishing Group Ltd ("BMJ") its licensees and where the relevant Journal is co-owned by BMJ to the co-owners of the Journal, to publish the Work in this journal and any other BMJ products and to exploit all rights, as set out in our licence.

The Submitting Author accepts and understands that any supply made under these terms is made by BMJ to the Submitting Author unless you are acting as an employee on behalf of your employer or a postgraduate student of an affiliated institution which is paying any applicable article publishing charge ("APC") for Open Access articles. Where the Submitting Author wishes to make the Work available on an Open Access basis (and intends to pay the relevant APC), the terms of reuse of such Open Access shall be governed by a Creative Commons licence – details of these licences and which Creative Commons licence will apply to this Work are set out in our licence referred to above.

Other than as permitted in any relevant BMJ Author's Self Archiving Policies, I confirm this Work has not been accepted for publication elsewhere, is not being considered for publication elsewhere and does not duplicate material already published. I confirm all authors consent to publication of this Work and authorise the granting of this licence.

For peer review only

Electronic cigarettes as a smoking cessation aid for cancer patients: beliefs and behaviours of clinicians in the UK

Brett J[1], Davies EL[1], Matley F[1], Aveyard P[2], Wells M[3], Foxcroft D[1], Nicholson B[2], De Minor-Silva S[4], Sinclair L[5], Jake S[6], Watson E[1]

[1]Oxford Brookes University
[2]University of Oxford
[3]Imperial College Healthcare NHS Trust
[4]Oxford University Hospitals NHS Foundation Trust
[5]University of Edinburgh
[6] Vaper Representative

Corresponding author: Brett J. JBrett@brookes.ac.uk
Funder: Cancer Research UK

Dr Jo Brett	Senior Research Fellow, Supportive Cancer Care, Health and Life Sciences, Oxford Brookes University
Dr Emma L Davies	Senior Lecturer in Psychology, Department of Psychology, Social Work and Public Health Oxford Brookes University
Fiona Matley	Research Assistant, Supportive Cancer Care , Health and Life Science, Oxford Brookes University
Prof. Paul Aveyard	Professor of Behavioural Medicine Nuffield Department of Primary Care Health Sciences, University of Oxford
Prof. Mary Wells	Lead Nurse for Research at Imperial College Healthcare NHS Trust and a Professor of Practice in Cancer Nursing at Imperial College, Faculty of Medicine, Department of Surgery & Cancer
Prof. David Foxcroft	Professor of Community Psychology and Public Health, Department of Psychology, Social Work and Public Health, Oxford Brookes University
Dr Brian Nicholson	Senior Clinical Researcher and Macmillan GP, Nuffield Department of Primary Care Health Sciences, University of Oxford
Dr Shiroma De Silva Minor	Consultant Clinical Oncologist, Oxford University Hospitals NHS Foundation Trust, Oxford
Lesley Sinclair	Research Fellow, Molecular, Genetic and Population Health Sciences, University of Edinburgh
Sarah Jakes	Vaper Representative
Prof Eila Watson	Prof of Supportive Cancer Care, Supportive Cancer Care, Health and Life Sciences, Oxford Brookes University

Word Count: 3536

Abstract:

Objectives: To explore UK clinicians' beliefs and behaviours around recommending e-cigarettes as a smoking cessation aid for cancer patients

Design: Cross-sectional online survey

Setting: England, Wales, Scotland and Northern Ireland

Participants: Clinicians involved in the care of patients with cancer

Primary and secondary outcomes: Behavioural Change Wheel COM-B, knowledge, beliefs, current practice around e-cigarettes and other smoking cessation practices

Method: Clinicians (n=506) completed an online survey to assess beliefs and behaviours around e-cigarettes and other smoking cessation practices for cancer patients. Behavioural factors associated with recommending e-cigarettes in practice were assessed.

Results: 29% of clinicians would not recommend e-cigarettes to cancer patients who continue to smoke. Factors associated with recommendation include smoking cessation knowledge (1.56, CI 1.01 - 2.44) and e-cigarette knowledge (OR =1.64, CI 1.06-2.55), engagement with patients regarding smoking cessation (OR =2.12, CI 1.12-4.03), belief in the effectiveness of e-cigarettes (OR =2.36 CI 1.61-3.47), and belief in sufficient evidence on e-cigarettes (OR = 2.08 CI 1.10-4.00) and how comfortable they felt discussing e-cigarettes with patients (OR = 1.57 CI 1.04-2.36).

Conclusion: Many clinicians providing cancer care to patients who smoke do not recommend e-cigarettes as a smoking cessation aid and were unaware of national guidance supporting recommendation of e-cigarettes as a smoking cessation aid.

For peer review only - http://bmjopen.bmj.com/site/about/guidelines.xhtml

Strengths and limitations of this study

- This study reports an online survey with a wide-range of clinicians to assess their beliefs and current practices around e-cigarettes as a smoking cessation aid for cancer patients.
- The survey used the COM-B behavioural model to understand the factors that influence clinicians to recommend or not recommend e-cigarettes.
- Quota sampling enabled representation across relevant clinical roles in primary and secondary care and across geographical areas in the UK
- The sample is limited to UK clinicians where public health policy supports use of e-cigarettes as a smoking cessation aid for risk reduction compared to tobacco cigarette smoking
- The sampling was not random and so participation could have been affected by whether clinicians were interested in the topic, although paying clinicians to complete the study aimed to mitigate this.

Key words: E cigarettes, cancer, smoking cessation, behavioural change, current practice

For peer review only - http://bmjopen.bmj.com/site/about/guidelines.xhtml

Background

Smoking is a well-established risk factor for many common cancers.[1-3] The adverse effects of smoking continue after a cancer diagnosis, increasing the risk of treatment-related complications, recurrence, the development of a second primary cancer, and mortality from both cancer-related and non–cancer-related causes.[4-11] Despite the increased risk of complications of cancer treatment, recurrence, and death, many patients with smoking-related cancers continue smoking following diagnosis[12-15] having tried and failed to stop smoking. Effective aids for cessation are available, but support to quit is not routinely offered as part of cancer care.[16,17] One study found that 39% (N=1129) of lung cancer patients, 37% (N=281) upper aero-digestive tract cancer patients, and 49% (N=850) of bladder cancer patients continued to smoke one year after diagnosis, figures that were likely to be higher as a third of the potential participants' smoking status was unknown.[18] To enhance the length and health-related quality of their lives, efforts are needed to support cancer patients to stop smoking cigarettes.

In recent years e-cigarettes have grown in popularity as a cessation aid among smokers worldwide, but the e-cigarette regulatory environment in each country varies considerably due to policy makers' reactions to a rapidly developing evidence base.[19,20] Often this is influenced by strategic positions on tobacco control e.g. harm prevention vs. harm reduction,[21] and whether e-cigarettes are classified as a tobacco, medicinal or consumer product.[19] The main debates focus on trying to achieve a balance between the risks and potential of e-cigarettes.[21]

For peer review only - http://bmjopen.bmj.com/site/about/guidelines.xhtml

Support for the use of e-cigarettes for smoking cessation is endorsed by several health-related organisations in the UK including Public Health England,[22] Cancer Research UK,[23] the National Health Service[24] and the National Centre for Smoking Cessation and Training.[25] Public Health Scotland,[26] Public Health Wales,[27] the British Medical Association,[28] the Royal College of General Practitioners[29] and the Royal College of Physicians[30] also acknowledge that e-cigarettes are considered less harmful than smoking tobacco cigarettes or that some people may find using e-cigarettes useful for stopping or reducing smoking.

In the UK and other European countries, the manufacture, presentation and sales of e-cigarettes are regulated by the Tobacco Products Directive (2014/40/EU).[31, 32] The regulation prohibits sales of e-cigarettes to people under 18 years, most forms of advertising and places restrictions on the type and quality of ingredients. In France there are further restrictions on the use of e-cigarettes in public places.[33] The regulatory environment differs substantially in other parts of the world.

In the United States, for instance, the Centres for Disease Control and Prevention (CDC) advice is not unlike many UK public health bodies in stating that "if (adults) choose to use e-cigarettes as an alternative to cigarettes, they should completely switch from cigarettes to e-cigarettes and not partake in an extended period of dual use of both products that delays quitting smoking completely".[34] The World Health Organisation position is that there is insufficient evidence concerning comparisons with combustible cigarettes or efficacy for smoking cessation, and that the use of e-cigarettes is harmful.[35], although In October, 2018, 72 experts with no connections to the tobacco industry wrote to the WHO Director-General

to argue that WHO should embrace innovation and more actively include tobacco harm reduction in its strategy to tackle the burden of smoking-related disease.

Whilst the need for further evidence around impacts of e-cigarettes on cessation and long-term harms is widely acknowledged, many health-related organisations also recognise that e-cigarettes may have a potential role in smoking cessation and could help people that may otherwise continue to smoke. A risk reduction policy to encourage smoking cessation is particularly important in people who have been diagnosed with cancer who continue smoking and therefore increase their risk of recurrence, other co-morbidities and premature death as a result. [4-11]

In the UK, e-cigarettes have swiftly become the most popular smoking cessation product for smokers.[36,37] There are 3.6 million EC users in the UK, of which only 0.8% have never smoked.[37] E-cigarettes are used in 30% of quit attempts with currently around 20% of smokers and 30% of recent ex-smokers using them.[38] Evidence shows that e-cigarettes help smokers to stop smoking long-term[39-41] and a recent study suggests that e-cigarettes are more effective for smoking cessation than nicotine-replacement therapy, when both products are accompanied by behavioural support.[42] However, qualitative evidence implies that clinicians may be uncertain about the use of e-cigarettes as a cessation aid, with some expressing hostility and reporting practices that are not consonant with the evidence.[43]
The aims of this study were to understand clinicians' beliefs and behaviours related to e-cigarettes for patients with cancer who continue to smoke, and to understand the behavioural factors that may promote or inhibit recommending e-cigarettes.

Methods

Design

The study was a cross-sectional online survey of clinician's knowledge, beliefs and current practice of smoking cessation and e-cigarettes. The survey was sent to clinicians involved in the adult cancer care pathway, working in primary and secondary care.

Survey development

The survey was developed using methods suggested by Bowling (1997).[44] The questions drew on 1) a literature review to identify evidence of clinicians' knowledge, attitudes, behaviours and current practice with respect to smoking cessation interventions, including e-cigarettes, in cancer patients; 2) expert opinion; 3) drawing on Behaviour Change Wheel (BCW).[45,46] Development also drew on previous survey questions exploring attitudes to e-cigarettes.[47-49] The face validity of the survey was evaluated with five general practitioners (GPs) and five cancer clinicians to evaluate whether the survey was appropriate and the questions were understood. This resulted in minor modification of wording of some questions.

The final questionnaire was structured according to the COM-B behaviour model based on Michie's Behaviour Change Wheel for development of interventions.[45,46] This model proposes that people need capability (C), opportunity (O) and motivation (M) to perform a behaviour (B) and was developed to guide understanding of behaviour in context and develop behavioural targets. The model proposes that for someone to engage in a particular behaviour at a given moment they must be physically able and have the psychological ability and have the social and physical opportunity to enact the behaviour

For peer review only - http://bmjopen.bmj.com/site/about/guidelines.xhtml

and, in addition, want or need to enact the behaviour more than any other competing behaviours at that moment. This inclusive definition of motivation covers basic drives and automatic processes such as habit and impulses as well as reflective processes including intention and choice. If a desired behaviour is not occurring (or an undesirable behaviour occurring) then an analysis of the determinants of the behaviour will help to define what needs to shift in order for the desired behaviour to occur (or the unwanted behaviour to cease).

The questionnaire included items relating to psychological capability (knowledge of smoking status of patients, knowledge and skills about smoking cessation in general, knowledge and skills about of e-cigarettes,); physical opportunity (time constraints talking to patients about e-cigarettes, physical constraints due to policy); social opportunity (relationship with patient and how this impacts on smoking cessation advice given, social norms e.g. most my colleagues support use of e-cigarettes, most my colleagues feel uncomfortable recommending e-cigarettes, clinicians should discourage smoking); reflective motivation (motivation to engage in smoking cessation with cancer patients, beliefs about how effective e-cigarettes are for cancer patients, beliefs about the clinician's role, beliefs about the evidence base around e-cigarettes, attitudes towards e-cigarettes, beliefs about the harms of e-cigarettes particularly in comparison to tobacco cigarettes, and automatic motivation (how comfortable they feel giving smoking cessation advice in general and in giving specific advice on e-cigarettes to patients). A full copy of the questionnaire is available electronically (insert link here)

For peer review only - http://bmjopen.bmj.com/site/about/guidelines.xhtml

Sample:

The survey was distributed electronically by M3, the research arm of Doctors.net.uk, a leading market research consultancy specialising in high-quality online research using pre-recruited panels of medical professionals. All clinicians who are registered with doctors.net.uk have to provide their clinical registration number during the registration process. M3 have 7781 GPs, 436 oncologists, 708 surgeons, 221 cancer nurse specialists, and 315 practice nurses on their research panels. Sampling was restricted to currently practising clinicians, was stratified by NHS region, and was conducted on a 'first come, first served' basis; the target number of responses was 100 for each of the clinician types. When the quota of responses from each type of clinician or from each UK NHS region was reached, the survey was closed for that clinician group or region.

The survey was completed between Nov 2018 and February 2019. A small financial incentive was offered. Participants confirmed they had read the participant information and consented to take part via email before the survey link was sent.

Analyses

Anonymised electronic responses were imported into SPSS (version 25) for analysis. Frequencies and proportions were used to summarise questionnaire responses. Results are reported using the COM-B model: physical and psychological capability; physical and social opportunity; and automatic and reflective motivation

Means and standard deviations for each measure for the whole sample were calculated and then compared by clinician occupation. One-way ANOVA was used to compare means for

the other measures. Multiple comparisons were taken into account using a Bonferroni correction. Student-Newman-Keuls post-hoc tests were used to examine differences reported.

Chi squared tests were used to compare the clinicians on categorical measures. Binomial logistic regression models were used to predict the likelihood of recommending e-cigarettes to cancer patients. The dependent variable was dichotimised by always/nearly always /often recommend e-cigarettes (Q5_3) vs. sometimes/ infrequently/ never.

Ethics:

This study was approved by the Oxford Brookes University Research Ethics Committee (2017 44 Brett)

Patient and Public Involvement:

Cancer service users and vapor representatives were involved in the proposal development, questionnaire development and dissemination of the results of this study. One vapor representative was involved in the write up and is an author on the paper.

Results:

Characteristics of respondents

The online survey was completed by 506 clinicians: 103 GPs, 102 oncologists, 100 cancer surgeons, 102 practice nurses and 99 cancer nurse specialists (CNSs). One CNS was excluded because they were a CNS for children. Table 1 describes the clinicians' characteristics:

Table 1 Clinician Characteristics

**Demographics**	All participants % (N = 506)	Primary Care % (n = 205)	Secondary Care % (n = 301)
Gender			
Male	41.1	35.1	45.2
Female	57.5	63.9	53.2
Prefer not to say	1.4	1.0	1.7
Years of professional experience	1995 (1968-2009)	1994	1996
Role			
General practitioner (GP)	20.4	40.5	
Practice nurse	20.2		
Cancer surgeon	19.8		59.5
Oncologist	20.2		
Cancer nurse specialist	19.6		
NHS region			
London	15.8	12.2	18.3
South of England	21.1	21.5	20.9
Midlands & East SEA	25.5	25.4	25.6
North of England	20.6	22.9	18.9
Scotland	12.1	13.7	11.0
Wales	2.2	2.4	2.0
Northern Ireland	2.8	2.0	3.3
Main cancer group they care for			
All	37.0	76.1	10.3
Breast	24.1	12.2	32.2
Prostate	19.4	13.7	23.3
Lung / mesothelioma	18.0	11.7	22.3

For peer review only - http://bmjopen.bmj.com/site/about/guidelines.xhtml

Bowel	11.9	5.4	16.3
Kidney	11.9	0.5	19.6
Bladder	11.3	0.5	18.6
Other cancer groups	< 10	<5	<15
Smoking status of clinicians			
Smoke tobacco cigarettes	2.0	1.5	2.3
** Use e-cigarettes	1.2	1.5	1.0
Ex-smoker	21.3	22.9	20.3
Never smoker	72.3	72.2	72.4
Other	1.4	1.5	1.3
Prefer not to say	3.6	2.4	4.3

Reported behaviour:

Less than a third (29% n=147) of clinicians would not recommend e-cigarettes to cancer patients who smoke. Just over half (51%, n=258) would recommend e-cigarettes as an interim measure, to help patients stop smoking completely, while 20% (n=101) would recommend e cigarettes as a partial replacement for smoking tobacco.

Psychological capability

Most clinicians (78%, n=394) knew the smoking status of their cancer patients, and routinely recorded their smoking status (73% n=368). Sixty seven percent (n=339) reported that they routinely recommended patients stop smoking, or cut down (52%, n=263). Twenty-nine percent (n=147) referred patients to the NHS stop smoking services, 14% (n=71) recommended NRT, 9% (n=46) recommended digital smoking cessation tools, and 5% (n=25) prescribed medication (Varenicline or Bupropion).

For peer review only - http://bmjopen.bmj.com/site/about/guidelines.xhtml

Many clinicians felt they had insufficient knowledge (57%, n=286) and training (73%, n=370) to provide advice about e-cigarettes to patients and a further 36% (n=182) indicated that they did not know the efficacy of e-cigarettes with regard to smoking cessation. Clinicians derived information about e-cigarettes from several sources. Overall, only 9.5% (n=48) of clinicians knew whether their organisation had guidance concerning advice to patients on e-cigarette use. Most clinicians had sought information about e-cigarettes from government/health agencies (55%), but also from professional associations (37%), healthcare colleagues (29%), news/media/advertising (24%), scientific literature (23%), professional development/training (22%) and charities (18%). Nineteen percent of clinicians had never sought information about e-cigarettes. One quarter of respondents (25%, n=124) were uncertain whether e-cigarettes were less harmful than smoking tobacco, while 10% (n=52) thought e-cigarettes were equally harmful or more harmful than smoking tobacco. Eighteen percent (n=93) considered using e-cigarettes to be more harmful than regular nicotine replacement therapies (e.g. gum, nasal spray, patches) and 54% (n=273) were uncertain.

Physical opportunity

The majority of clinicians (56%, n=285) said that e-cigarette use was prohibited in all areas at their main place of work. Twenty three percent (n=119) reported that the use of e-cigarettes was permitted, with 24% (n=29) reporting use in designated smoking areas only. Overall, 51% (n=258) agreed that time constrained their ability to talk about e-cigarettes with patients.

For peer review only - http://bmjopen.bmj.com/site/about/guidelines.xhtml

Social opportunity

The nature of the relationships between clinicians and patients with cancer were important in whether and how smoking cessation was discussed. Fifty five percent (n=278) of clinicians reported that having a good relationship would make them more likely to speak to their patient about stopping or cutting down, while 45% (n=228) reported that having a poor relationship would make them less likely to discuss smoking cessation. Many clinicians (42% n= 212) felt uncomfortable when asked by patients for an opinion on e-cigarettes. The large majority, 82%, had been asked about e-cigarettes by patients in the past year (2017/18), up from 21% in 2016/17.

Thirty eight percent (n=192) said that most of their colleagues would feel uncomfortable recommending e-cigarettes to patients with cancer, and 37% (n=187) were unsure whether clinicians should discourage patients with cancer from using e-cigarettes.

Automatic motivation

Subconscious biases towards e-cigarettes were influenced by clinicians' beliefs around the effectiveness of and evidence on e-cigarettes, as reported in the psychological capability section above.

Clinicians reported that their decisions to speak with patients with cancer about smoking cessation were influenced by their perceptions of the patient. Clinicians reported that they were more likely to discuss smoking cessation if they judged the person was motivated to quit (69%, n=349), or was coping well (67%, n=339).

Reflective Motivation

Reflective motivation related to the clinicians' perceived role in smoking cessation and national and organisational policy on e-cigarettes. Two thirds (65%, n=327) of clinicians agreed that they should play a greater role in helping cancer patients stop smoking. Clinicians were divided over whether e-cigarettes should be licensed and available on prescription for patients with cancer, with 39% (n=199) respondents disagreeing, and 32% (n=162) saying they should be available on prescription. Furthermore, 30% (n=150) of clinicians felt that public health campaigns, such as Stoptober should not endorse using e-cigarettes as a way to give up smoking tobacco, while 29% (n=149) thought e-cigarettes should be endorsed in campaigns.

Differences in e-cigarettes practice between clinicians

GPs and practice nurses were significantly more likely to say that they recommended e-cigarettes to cancer patients than the other clinicians included in the study (see Table 2). GPs and practice nurses also rated their knowledge about e-cigarettes higher than did specialist cancer care clinicians, and were also more likely to report having sufficient time to discuss smoking with patients and rated their role in helping patients cut down smoking as more important.

Practice nurses engaged in significantly more behaviours (e.g. ascertaining smoking status, advising patients, supporting e-cigarette use in patients) related to smoking cessation with patients than all the other groups. GPs engaged in significantly more behaviours than the other clinicians, but fewer than practice nurses. Practice nurses were significantly more

For peer review only - http://bmjopen.bmj.com/site/about/guidelines.xhtml

likely to believe in the effectiveness of e-cigarettes in helping cancer patients stop smoking compared to other clinicians.

For peer review only

For peer review only - http://bmjopen.bmj.com/site/about/guidelines.xhtml

Table 2: **COM-B measures by HP– Mean and SD**

	Total	General Practitioner	Practice Nurse	Cancer Surgeon	Oncologist	Cancer Nurse Specialist	Test statistic, p
N of participants	506	103	102	100	102	99	
Recommend e-cigarettes always/often, N (%)	97 (19.2)	24 (23.3%)	36 (35.3)	9 (9)	16 (15.7)	12 (12.1)	χ^{2} = 28.90, p<.001
**Capability**							
Know status	4.72 (.59)	4.75 (.48)	4.78 (.46)	4.81 (.46)	4.75 (.60)	4.52 (.81)	F = 4.17, p=.002*
General smoking knowledge	3.40 (1.03)	3.80 (.86)	3.75 (1.08)	3.10 (1.00)	3.17 (1.03)	3.19 (.95)	F = 12.09, p<.001*
E-cig knowledge	2.34 (.99)	2.40 (.89)	2.45 (1.07)	2.21 (.94)	2.54 (1.05)	2.11 (.95)	F = 3.32, p=.011
**Opportunity**							
Time	2.60 (1.03)	2.46 (.95)	2.87 (.94)	2.39 (1.00)	2.29 (1.06)	3.02 (1.00)	F= 10.47, p<.001*
Patient relationships	2.51 (.68)	2.45 (.72)	2.52 (.66)	2.59 (.64)	2.49 (.67)	2.52 (.72)	F = .59, p=.668
Social norms	3.00 (.72)	2.98 (.86)	3.18 (.75)	2.85 (.66)	3.08 (.65)	2.91 (.64)	F =3.36, p=.010
**Motivation**							
Engagement with patients who smoke	2.77 (.61)	2.90 (.55)	3.21 (.55)	2.60 (.54)	2.55 (.58)	2.59 (.55)	F= 26.13, p<.001*
Effectiveness in helping cancer pts	2.83 (1.50)	2.86 (1.48)	3.39 (1.43)	2.49 (1.54)	2.81 (1.45)	2.57 (1.44)	F = 5.89, p<.001*
Importance of HP role	4.35 (.76)	4.48 (0.62)	4.60 (.60)	4.33 (.83)	4.20 (.81)	4.15 (.84)	F =6.40, p<.001*
Lack of evidence N (%)	242 (47.8)	57 (55.3)	51 (50)	44 (44)	41 (40.2)	50 (50.5)	χ^{2} =5.60 p=.231
Attitudes	3.22 (.61)	3.23 (.64)	3.39 (.62)	3.08 (.61)	3.26 (.53)	3.13 (.59)	F =.4.04, p=.003
Harm	3.16 (.68)	3.25 (.63)	3.15 (.67)	3.18 (.66)	3.22 (.71)	3.01 (.71)	F =1.32, p=.263

Better than smoking	3.86 (.96)	4.00 (1.00)	3.84 (1.00)	3.82 (.81)	4.07 (.86)	3.56 (1.05)	F = 4.39, p=.002*
Comfortable discussing smoking in general	4.40 (.76)	4.45 (.75)	4.44 (.71)	4.49 (.72)	4.39 (.81)	4.24 (.80)	F =1.59, p=.175
Comfortable discussing e-cigs	2.91 (1.16)	3.09 (1.13)	3.04 (1.23)	2.65 (1.17)	3.00 (1.11)	2.76 (1.12)	F =2.79, p=.026

Note * = significant for F tests when multiple comparisons taken into consideration, accepted p value corrected to 0.003

For peer review only - http://bmjopen.bmj.com/site/about/guidelines.xhtml

For peer review only

For peer review only - http://bmjopen.bmj.com/site/about/guidelines.xhtml

Logistic regression model

Table 3 presents a logistic regression model including all COM-B factors and controlling for clinician type. In this model, not recommending e-cigarettes is associated with a lack of knowledge regarding smoking cessation (OR = 1.56, CI 1.01 - 2.44) and e-cigarettes (OR =1.64 CI 1.06-2.55). Additionally, greater engagement with patients regarding smoking cessation (OR =2.12 CI 1.12-4.03), belief in the effectiveness of e-cigarettes (OR =2.36 CI 1.61-3.47), belief in sufficient evidence on e-cigarettes (OR = 2.08 CI 1.10-4.00) and the social opportunity factor of how comfortable they felt discussing e-cigarettes with patients (OR = 1.57 CI 1.04-2.36) all significantly predicted recommending e-cigarettes to cancer patients. Specifically, those who reported higher levels of engagement around smoking cessation, and those who were comfortable discussing e-cigarettes were more likely to recommend them. However, those who felt the evidence base was lacking were less likely to recommend them.

w only

For peer review only - http://bmjopen.bmj.com/site/about/guidelines.xhtml

Table 3: Results of full binary logistic regression model exploring all factors relating to capability, opportunity and motivation to recommend e-cigarettes to cancer patients

				95% CI for Odds Ratio		
	B	Wald(df =1)	p	Lower	Odds Ratio	Upper
**Capability**						
Know status	0.758	3.320	0.068	0.944	2.134	4.821
General smoking knowledge	-0.440	3.866	0.049	1.010	1.563	2.439
E-cig knowledge	0.495	4.878	0.027	1.057	1.641	2.546
**Opportunity**						
Time	0.083	0.237	0.626	0.778	1.087	1.517
Patient relationships	-0.092	0.128	0.720	0.551	0.912	1.510
Social norms	-0.047	0.028	0.867	0.549	0.954	1.658
**Motivation**						
Engagement with patients who smoke	0.752	5.308	0.021	1.119	2.122	4.025
Effectiveness in helping cancer pts	0.858	19.09	0.000	1.605	2.359	3.466
Importance of HP role	0.259	0.834	0.361	0.743	1.295	2.257
Sufficient evidence	-0.745	4.963	0.026	1.099	2.083	4.000
Attitudes	0.370	1.038	0.308	0.711	1.447	2.947
Harm	0.023	0.006	0.941	0.556	1.023	1.885
Better than smoking	-0.009	0.001	0.972	0.593	0.991	1.655
Comfortable discussing smoking in general	-0.402	2.180	0.140	0.392	0.669	1.141
Comfortable discussing e-cigs	0.448	4.594	0.032	1.039	1.565	2.356
**Health professional type**						
		5.385	0.250			
GP	0.276	0.256	0.613	0.453	1.318	3.839
Practice Nurse	0.281	0.294	0.588	0.479	1.324	3.659
Cancer Surgeon	-0.805	1.791	0.181	0.138	0.447	1.453
Oncologist	-0.391	0.514	0.473	0.232	0.676	1.971
**Constant**	-11.416	20.331	0.000		0.000	

Note: reference categories - for HP type = Cancer Nurse Specialist, for sufficient evidence = yes

For peer review only - http://bmjopen.bmj.com/site/about/guidelines.xhtml

2.08 CI 1.10-4.00

For peer review only

For peer review only - http://bmjopen.bmj.com/site/about/guidelines.xhtml

For peer review only

For peer review only - http://bmjopen.bmj.com/site/about/guidelines.xhtml

Discussion

The findings from this study suggest relatively low levels of clinician support around recommending e-cigarettes to cancer patients. Despite a growing evidence base to support use and popularity of e-cigarettes as a smoking cessation aid in the UK, nearly a third of clinicians do not recommend e-cigarettes to cancer patients who smoke. Not recommending e-cigarettes is associated with a lack of knowledge regarding smoking cessation and e-cigarettes, lack of engagement in smoking cessation practices with patients that smoke, low belief in effectiveness of e-cigarettes, low belief in evidence around e-cigarettes, and not feeling comfortable discussing e-cigarettes with their patients.

In line with the results of this study, a survey of 124 members of The British Thoracic Oncology Group in April 2015 showed that 93% of clinicians agreed that they needed more information and guidance on e-cigarettes to advise patients. Clinicians lacked confidence to advise patients with lung cancer to use e-cigarettes.[47] Our findings indicate that, three years after this study, clinicians continue to lack knowledge and confidence in recommending e-cigarettes to cancer patients despite a UK public health policy to support e-cigarettes. Clinicians from various specialties have reported a need for training and local guidance around e-cigarettes in line with the national public health policy.[50,51] Interventions are needed to target the reported behavioural factors associated with clinicians' reluctance to recommend e-cigarettes.

This study highlights that the COM-B components of psychological capability, social opportunity and automatic and reflective motivation were all associated with clinicians' beliefs and behaviours around recommending e-cigarettes. Improving knowledge through accessible training on e-cigarettes alongside local adoption of public health policies around

e-cigarettes throughout the NHS may support clinicians to feel more confident and comfortable in recommending them to patients. The National Institute for Health and Care Excellence (NICE) in the UK advise that clinicians have an informed discussion with patients on use of e-cigarettes to stop smoking.[52]

A recent study reports that practitioners suggested the development of decision aids around e-cigarettes, such as a leaflet, booklet or online resource to use during consultations with patients.[53] This would aid a more 'neutral' decision around use of e-cigarettes and potentially improve confidence around discussions on e-cigarettes.[54] Furthermore, engagement from local clinical commissioning groups and clinical leads alongside accessible training may support clinicians in providing advice on e-cigarettes to cancer patients.
To date there is no medicinally licenced EC in the UK, or anywhere else in the world, which is possibly presenting challenges for clinicians wishing to demonstrate e-cigarettes or recommend for inpatient use. In this study, clinicians were divided over this decision, with two fifths of clinicians not supportive, and nearly a third supportive of licensing e-cigarettes for medicinal uses. Clinicians may be more comfortable recommending e-cigarettes if they were available on prescription.

Debate is also needed on how e-cigarettes could be integrated into smoking cessation practices delivered by clinicians to patients with cancer. Smoking cessation practices are already well developed in other disease groups such as coronary heart disease (CHD).[55] It is therefore timely to examine the role of a smoking cessation service within the cancer pathway, including the role of e-cigarettes can play in helping cancer patients to quit smoking long term after a diagnosis.[56] While attendance at a cancer clinic provides an

For peer review only - http://bmjopen.bmj.com/site/about/guidelines.xhtml

opportunity for clinicians to provide smoking cessation support to those who smoke, this is not currently routinely offered. Patients with cancer have highlighted the need for smoking cessation support, and have reported difficulty in attending external smoking cessations services in addition to all their other clinic appointments.[12] Smoking cessation advice has historically been the role of primary care or community smoking cessation services, and this is reflected in the greater knowledge, confidence and more positive attitude towards e-cigarettes among practice nurses and GPs than among cancer specialists reported in this study. However, lack of funding has seen a decline in the smoking cessation services, and alternatives are needed. Clinicians worry that discussing smoking may damage the relationship with the patient, which is essential for the often onerous treatment needed for cancer.[57] Clinicians also seem to believe that patients cannot stop smoking.[58]

Strengths and limitations

To our knowledge, this study is the first to examine a broad range of clinicians' behaviours and beliefs around the use of e-cigarettes in patients across different cancer groups. The COM-B model has previously been used to develop smoking cessation interventions.[59] In this study, it has enabled the identification of factors, which could be used to improve clinicians' recommendation of e-cigarettes.

The recruitment procedure for this survey utilised an existing network of electronically active clinicians from the research arm of doctors.net.com. This method has the advantage of speed and guaranteed response which is beneficial considering that surveys with busy clinicians have commonly suffered from poor response rates. However, this sample may not be representative of the population of clinicians in the United Kingdom. Quota sampling

For peer review only - http://bmjopen.bmj.com/site/about/guidelines.xhtml

ensured diversity in our sample, which included clinicians who worked with patients who had a wide range of cancer diagnoses, and not just those with cancers directly associated with smoking. However, in quota sampling, the sample has not been chosen using random selection

The potential for response bias should be considered; for example, those with a greater interest in smoking cessation for cancer patients may have been more likely to respond, and the incentive may have encouraged participation, whereas clinicians who are smokers may have been under-represented. In addition, findings rely on self-report.

Conclusions

Despite the evidence that e-cigarettes help smokers quit smoking, and the positive public health stance towards e-cigarettes in the UK, clinicians remain cautious about recommending e-cigarettes to cancer survivors who continue to smoke. Clinicians require training and support on how to integrate e cigarettes in smoking cessation advice for cancer patients and adoption of the UK evidence based guidance at regional and local level is needed.

For peer review only - http://bmjopen.bmj.com/site/about/guidelines.xhtml

Acknowledgements

This work was funded and supported by Cancer Research UK. We thank the clinicians who participated in this online survey, and MedeConnect Healthcare Insight, the research arm of Doctors.Net.UK for recruiting, sending and collating results for the online survey.

Summary results have been presented at National Cancer Research Institute (NCRI) conference Glasgow Nov 2018, and International Psycho-Oncology Society (IPOS) conference in Banff, Canada, Sept 2019

E-cigarettes as a Smoking Cessation aid in Cancer Patients: Clinicians knowledge, attitude and current practice. NCRI 2018 abstract 2171

E-cigarettes as a Smoking Cessation aid in Cancer Patients: Clinicians knowledge, attitude and current practice. IPOS 2019 abstract 751. Journal of Psychosocial Oncology – Research and Practice Sept 2019, vol 1: suppl 1 1S

Author Contributions

JB, ED, EW, PA, FM, DF, & MW conceived and designed the study. Additionally, LS, BN, SDM, and SJ commented on the final funding application before submission. FM, JB & ED developed the initial questionnaire, with e-cigarette knowledge input from PA, SJ &LS, primary health care input from PA & BN, nursing input from MW & surgeon and oncology input from SDM. ED and FM conducted the statistical analysis, which was reviewed by DF. JB & FM wrote the initial manuscript. EW, PA, ED, DF & MW critically reviewed the initial manuscript, while LS, BN, SDM & SJ reviewed and commented on the second version of the manuscript. All authors read and approved the final manuscript.

Competing interests: The authors have no conflict of interest or disclosures. All authors declare no financial or other relationships or activities that could appear to have influenced the submitted work.

For peer review only - http://bmjopen.bmj.com/site/about/guidelines.xhtml

Ethical approval:

This study complies with ethical standards. Informed consent was gained. The study obtained ethics approval to conduct the study from Oxford Brookes University Ethics Committee (2017 44 Brett) and was performed in accordance with the Declaration of Helsinki.

Funder:

Cancer Research UK Ref: A24355 Dr Jo Brett. PA is an NIHR senior investigator and funded by the NIHR Oxford Biomedical Research Centre and the Applied Research Centre.

Data sharing/Data availability:

Data will be deposited in the university repository, where reasonable requests for access can be submitted. Data access can be requested from the corresponding author. The data will be kept for a minimum of ten years following publication.

For peer review only - http://bmjopen.bmj.com/site/about/guidelines.xhtml

References

1. Ordenez-Mena JM, Schottker B, on behalf of the Consortium on Health and Ageing: Network of Cohorts in Europe and the United States (CHANCES). Quantification of the smoking-associated cancer risk with rate advancement periods: meta-analysis of individual participant data from cohorts of the CHANCES consortium *BMC Medicine 2016*, 14 (62)
2. US Health and Human Resources. The Health Consequences of Smoking—50 Years of Progress: A Report of the Surgeon General 2016
3. US Health and Human Resources How Tobacco Smoke Causes Disease: The Biology and Behavioral Basis for Smoking-Attributable Disease A Report of the Surgeon GeneralAtlanta (GA): Centers for Disease Control and Prevention (US); 2010.
4. Yang BM Jacobs EJ, Gapstur SM, Stevens V, Cambell PT. Active Smoking and Mortality Among Colorectal Cancer Survivors: The Cancer Prevention Study II Nutrition Cohort. *J Clin Oncol* 2015, 33, 10.1200/JCO.2014.58.3831
5. Warren GW, Kasza KA, Reid ME, Cummings KM, Marshall JR. Smoking at diagnosis and survival in cancer patients. *Int J Cancer* 2013, 132,401–410.
6. Gajdos C, Hawn MT, Campagna EJ, Henderson WG, Singh JA, Houston T. Adverse effects of smoking on postoperative outcomes in cancer patients. *Ann Surg Oncol* 2012, 19,1430–1438
7. Gillison ML, Zhang Q, Jordan R, Xiao W, Westra WH, Trotti A, Spencer S, Harris J, Chung CH, Ang K. Tobacco smoking and increased risk of death and progression for patients with p16-positive and p16-negative oropharyngeal cancer. *J Clin Oncol* 2012, 30,2102–2111
8. Kenfield SA, Stampfer MJ, Chan JM, Giovannucci E. Smoking and prostate cancer survival and recurrence. *JAMA* 2011 305,2548–2555
9. Parsons A, Daley A, Begh R, Aveyard P. Influence of smoking cessation after diagnosis of early stage lung cancer on prognosis: systematic review of observational studies with meta-analysis. *BMJ* 2010, 340-348
10. Bittner N, Merrick GS, Galbreath RW, Butler WM, Wallner KE, Allen ZA, Brammer SG, Moyad M. Primary causes of death after permanent prostate brachytherapy. *Int J Radiat Oncol Biol Phys* 2008, 7,:433–440

For peer review only - http://bmjopen.bmj.com/site/about/guidelines.xhtml

11. Hooning MJ, Botma A, Aleman BM, Baaijens MH, Bartelink H, Klijn JG, Taylor CW, van Leeuwen FE. Long-term risk of cardiovascular disease in 10-year survivors of breast cancer. *J Natl Cancer Inst* 2007, 99,365–375
12. Wells M, Aitchison P, Harris F, Ozakinci G, Radley A, Bauld, L.. Williams, B.. Barriers and facilitators to smoking cessation in a cancer context: A qualitative study of patient, family and professional views. *BMC Cancer* 2017, 17(1), 348. doi: 10.1186/s12885-017-3344-z
13. Warren GW, Marshall JR, Cummings KM, Toll B, Gritz ER, Hutson A, Dibai S, Herbst R, Dresler C IASLC Tobacco Control and Smoking Cessation Committtee. Practice patterns and perceptions of thoracic oncology providers on tobacco use and cessation in cancer patients*. J Thorac Oncol* 2013,8(5),543–548
14. Park ER, Japuntich S, Temel J, et al. A smoking cessation intervention for thoracic surgery and oncology clinics: a pilot trial. *J Thorac Oncol* 2011, 6,1059–1065
15. Cooley M E, Sarna L., Kotlerman ., Lukanich JM, Jaklitsch M, Green S.B, Bueno R. Smoking cessation is challenging even for patients recovering from lung cancer surgery with curative intent. *Lung Cancer* 2009, 66(2), 218-225
16. Warren GW, Marshall JR, Cummings KM, Zevon MA, Reed R, Hysert P, Mahoney MC, et al. Automated tobacco assessment and cessation support for cancer patients. *Cancer* 2014,120(4),562–569
17. Murray R, Leonardi-Bee J, Marsh J, Jayes L, Britton J. Smoking status ascertainment and interventions in acute medical patients. *Clin Med* 2012,12,59-62.
18. Koshiaris C, Aveyard P, Oke J, Ryan R, Szatkowski L, Stevens R, Farley A. Smoking cessation and survival in lung, upper aero-digestive tract and bladder cancer: cohort study. *British Journal of Cancer* 2017, 117(8),1224-12
19. Kennedy RD, Awopegba A, De León E, et al. Global approaches to regulating electronic cigarettes. *Tobacco Control* 2017;26:440-445.
20. Bhalerao, A., Sivandzade, F., Archie, S.R. et al. Public Health Policies on E-Cigarettes (2019). *Curr Cardiol Repn* 2019;21:111. https://doi.org/10.1007/s11886-019-1204-y
21. Bold KW, Krishnan-Sarin S. E-cigarettes: Tobacco policy and regulation. *Current Addiction Reports* 2019;6(2):75-85. DOI: 10.1007/s40429-019-00243-5.

For peer review only - http://bmjopen.bmj.com/site/about/guidelines.xhtml

22. McNeill, A., Brose, L.S., Calder, R., Bauld, L., and Robson, D. Vaping in England: an evidence update including mental health and pregnancy, March 2020: a report commissioned by Public Health England. London: Public Health England 2020.
23. Cancer Research UK. E-cigarette Policy Statement. 2019. https://www.cancerresearchuk.org/sites/default/files/e-cigarette_policy_statement_oct_2019.pdf (accessed 23 June 2019).
24. NHS (National Health Service). Using e-cigarettes to stop smoking. 2020. https://www.nhs.uk/live-well/quit-smoking/using-e-cigarettes-to-stop-smoking/ (accessed 23 June 2020).
25. NCSCT (National Centre for Smoking Cessation and Training). E-cigarettes: a guide for healthcare professionals. 2020. https://elearning.ncsct.co.uk/e_cigarettes-stage_1 (accessed 23 June 2020).
26. Public Health Scotland. E-cigarettes. 2020. healthscotland.scot/health-topics/smoking/e-cigarettes (accessed 23 June 2020).
27. Public Health Wales. E-cigarettes (Electronic Nicotine Delivery Systems (ENDS)). 2017. http://www.wales.nhs.uk/sitesplus/888/opendoc/317614 (accessed 23 June 2020).
28. BMA (British Medical Association). E-cigarettes: balancing risks and opportunities. 2020. https://www.bma.org.uk/what-we-do/population-health/drivers-of-ill-health/e-cigarettes-balancing-risks-and-opportunities (accessed 23 June 2020).
29. [11] RCGP (Royal College of General Practitioners). RCGP Position Statement on the use of electronic nicotine vapour products (E-Cigarettes). 2017. https://www.rcgp.org.uk/policy/rcgp-policy-areas/e-cigarettes-non-combustible-inhaled-tobacco-products.aspx (accessed 23 June 2020).
30. [12] RCP (Royal College of Physicians). RCP advice on vaping following reported cases of deaths and lung disease in the US. 2019. https://www.rcplondon.ac.uk/projects/outputs/rcp-advice-vaping-following-reported-cases-deaths-and-lung-disease-us (accessed 23 June 2020).
31. [13] European Commission. The Tobacco Products Directive. 2014. https://ec.europa.eu/health/tobacco/products_en (accessed 23 June 2020).
32. [14] European Commission. Revision of the Tobacco Products Directive. 2016. https://ec.europa.eu/health/tobacco/products/revision_en (accessed 23 June 2020).

For peer review only - http://bmjopen.bmj.com/site/about/guidelines.xhtml

33. [15] Institute for Global Tobacco Control. Country Laws Regulating E-cigarettes: A Policy Scan. Baltimore, MD: Johns Hopkins Bloomberg School of Public Health. https://www.globaltobaccocontrol.org/e-cigarette_policyscan [Last Updated May 18, 2020] (accessed 23 June 2020).
34. [16] Centres for Disease Control and Prevention (CDC). About Electronic Cigarettes (E-Cigarettes). 2020. https://www.cdc.gov/tobacco/basic_information/e-cigarettes/severe-lung-disease.html (accessed 23 June 2020).
35. [17] World Health Organsition (WHO). Tobacco. 2020. https://www.who.int/news-room/fact-sheets/detail/tobacco (accessed 23 June 2020).
36. Health and Social Care Information Centre (HSCIC) (2019). Statistics on NHS Stop Smoking Services in England - April 2018 to March 2019 Aug, 2019
37. Action on Smoking and Health (ASH). Use of e-cigarettes (vaporisers) among adults in Great Britain September 2019 https://ash.org.uk/wp-content/uploads/2019/09/Use-of-e-cigarettes-among-adults-2019.pdf Accessed Nov 2019
38. NHS Smokefree report: https://www.nhs.uk/smokefree/help-and-advice/e-cigarettes Dec 2018. Accessed May 2019
39. McNeill A, Brose LS, Calder R, Bauld L & Robson D. Evidence review of e-cigarettes and heated tobacco products 2018. A report commissioned by Public Health England. London: Public Health England 2018
40. McRobbie H, Bullen C, Hartmann-Boyce J, Hajek P. Electronic cigarettes for smoking cessation and reduction. *Cochrane Database Syst Rev* 2014, (12), Cd010216. doi: 10.1002/14651858.CD010216.pub2
41. Hajek P, Etter, JF, Benowitz N, Eissenberg T, McRobbie H. Electronic cigarettes: review of use, content, safety, effects on smokers and potential for harm and benefit. *Addiction* 2014, 109(11), 1801-1810. doi: 10.1111/add.12659
42. Hajek P, Phillips-Waller A, Przulj D, Myers Smith K, Bisal N, Li J, Parrott S, Sasieni P, Dawkins L, Ross L, Wu Q, McRobbie H J. A Randomized Trial of E-Cigarettes versus Nicotine-Replacement Therapy *N Engl J Med* 2019, 380,629-637
43. Stepney M, Aveyard P, Begh R. GPs' and nurses' perceptions of electronic cigarettes in England: a qualitative interview study. *British Journal of General Practice* 2019, 69 (678), e8-e14.

For peer review only - http://bmjopen.bmj.com/site/about/guidelines.xhtml

44. Bowling A. Constructing and evaluating questionnaires for health services research. In: Research methods in health: investigating health and health services. Buckingham: Open University Press, 1997.

45. Michie S, van Stralen MM, West, R. (2011). The behaviour change wheel: A new method for characterising and designing behaviour change interventions. *Implementation Science* 2011, 6.doi: 10.1186/1748-5908-6-42

46. Michie S, Atkins L, West R. The Behaviour Change Wheel: A Guide to Designing Interventions. London: Silverback Publishing 2014. www.behaviourchangewheel.com

47. Sherratt FC, Newson L, Field JK. Electronic cigarettes: a survey of perceived patient use and attitudes among members of the British thoracic oncology group. *Respir Res* 2016;17:55.

48. Luxton NA, Hih P, Rahman MA. Electronic Cigarettes and Smoking Cessation in the Perioperative Period of Cardiothoracic Surgery: Views of Australian Clinicians *International Journal of Environmental Research and Public Health* 2018 15(11):248110.3390/ijerph15112481

49. Kanchastamsham V, Saladi S, Rodrigues J, Fernandes H, Patolia S, Santosh S. The knowledge, concerns and healthcare practices among physicians regarding electronic cigarettes. *J Community Hosp Intern Med Perspect*, 2017 Jul, 7(3), 144–150

50. Cummins S, Lieschow S, Bailey L, Bush T, Wassam K, Copeland L, Zhu SH. Knowledge and Beliefs about E cigarettes among Quitline Cessation Staff. *Addictive Behaviors*, 2016, 60,28-83

51. Stepney M, Aveyard P, Begh R. GPs and Nurses' perceptions of e-cigarettes in England. A Qualitative Study. *British Journal of General Practice* 2019, DOI: https://doi.org/10.3399/bjgp18X699821

52. Smoking cessation interventions and services [C] Evidence reviews for advice on e-cigarettes on general sale. National Institute for Health and Care Excellence (NICE) guideline NG92 Evidence reviews. March 2018.

For peer review only - http://bmjopen.bmj.com/site/about/guidelines.xhtml

https://www.nice.org.uk/guidance/ng92/evidence/c-advice-on-ecigarettes-on-general-sale-pdf-4788920848

53. Farley A, Koshiaris C, Oke J, Ryan R, Szatkowski L, Stevens R, Aveyard P. Physician Support of Smoking Cessation After Diagnosis of Lung, Bladder, or Upper Aerodigestive Tract Cancer. *Annals of family medicine* 2017 15(5), 443-450. doi: 10.1370/afm.2100

54. Butler CC, Pill R, Stott NCH. Qualitative study of patients' perceptions of doctors' advice to quit smoking: implications for opportunistic health promotion. *BMJ* 1998, 8,1878–1881

55. Farley A, Koshiaris C, Oke J, Ryan R, Szatkowski L Stevens R, Aveyard P (2015). Management of smoking in UK primary care in patients diagnosed with bladder, lung and head & neck cancer compared with coronary heart disease (CHD): a Clinical Practice Research Database (CPRD) study, European Journal of Cancer Care, 24, 46: 26

56. Wells M, Aitchison P, Harris F, Ozakinci G, Williams B, Entwistle V, Munro A, Radley A, Haw S, Culbard B, Bauld L (2015). Cancer diagnosis as an opportunity for increasing uptake of smoking cessation services among families: an exploratory study of patients', family members' and health professionals' views. Scottish Government Health Directorates Chief Scientist Office Report

57. Butler CC, Pill R, Stott NCH. Qualitative study of patients' perceptions of doctors' advice to quit smoking: implications for opportunistic health promotion. *BMJ* 1998, 8,1878–1881
58. Sheals K, Tombor I, McNeill A, Shahab L. A mixed method systematic review and meta analysis of mental health professionals' attitiudes towards smoking and smoking cessation among people with mental illnesses. Addiction 2016,111,1536-53
59. Gould GS, Bar-Zeev Y, Bovill M, Atkins L, Grupetta M, Clarke MJ, Bonevski B. Designing an implementation intervention with the Behaviour Change Wheel for health provider smoking cessation care for Australian Indigenous pregnant women. *Implementation Science* 2017, 12,114

For peer review only - http://bmjopen.bmj.com/site/about/guidelines.xhtml

STROBE Statement—Checklist of items that should be included in reports of ***cross-sectional studies***

	Item No	Recommendation	Page No
**Title and abstract**	1	(*a*) Indicate the study's design with a commonly used term in the title or the abstract	2
		(*b*) Provide in the abstract an informative and balanced summary of what was done and what was found	2
**Introduction**			
Background/rationale	2	Explain the scientific background and rationale for the investigation being reported	4
Objectives	3	State specific objectives, including any prespecified hypotheses	5
**Methods**			
Study design	4	Present key elements of study design early in the paper	5
Setting	5	Describe the setting, locations, and relevant dates, including periods of recruitment, exposure, follow-up, and data collection	6,7
Participants	6	(*a*) Give the eligibility criteria, and the sources and methods of selection of participants	6,7
Variables	7	Clearly define all outcomes, exposures, predictors, potential confounders, and effect modifiers. Give diagnostic criteria, if applicable	5,6
Data sources/ measurement	8*	For each variable of interest, give sources of data and details of methods of assessment (measurement). Describe comparability of assessment methods if there is more than one group	5
Bias	9	Describe any efforts to address potential sources of bias	5,6
Study size	10	Explain how the study size was arrived at	
Quantitative variables	11	Explain how quantitative variables were handled in the analyses. If applicable, describe which groupings were chosen and why	7
Statistical methods	12	(*a*) Describe all statistical methods, including those used to control for confounding	7
		(*b*) Describe any methods used to examine subgroups and interactions	7
		(*c*) Explain how missing data were addressed	N/A
		(*d*) If applicable, describe analytical methods taking account of sampling strategy	N/A
		(*e*) Describe any sensitivity analyses	N/A
**Results**			
Participants	13*	(a) Report numbers of individuals at each stage of study—eg numbers potentially eligible, examined for eligibility, confirmed eligible, included in the study, completing follow-up, and analysed	8
		(b) Give reasons for non-participation at each stage	N/A
		(c) Consider use of a flow diagram	N/A
Descriptive data	14*	(a) Give characteristics of study participants (eg demographic, clinical, social) and information on exposures and potential confounders	8 Table1
		(b) Indicate number of participants with missing data for each variable of interest	N/A
Outcome data	15*	Report numbers of outcome events or summary measures	8,9,10,11
Main results	16	(*a*) Give unadjusted estimates and, if applicable, confounder-adjusted estimates and their precision (eg, 95% confidence interval). Make	8,9,10,11

For peer review only - http://bmjopen.bmj.com/site/about/guidelines.xhtml

		clear which confounders were adjusted for and why they were included N/A	
		(*b*) Report category boundaries when continuous variables were categorized	N/A
		(*c*) If relevant, consider translating estimates of relative risk into absolute risk for a meaningful time period	N/A
Other analyses	17	Report other analyses done—eg analyses of subgroups and interactions, and sensitivity analyses	10,11
**Discussion**			
Key results	18	Summarise key results with reference to study objectives	12
Limitations	19	Discuss limitations of the study, taking into account sources of potential bias or imprecision. Discuss both direction and magnitude of any potential bias	14,15
Interpretation	20	Give a cautious overall interpretation of results considering objectives, limitations, multiplicity of analyses, results from similar studies, and other relevant evidence	12-14
Generalisability	21	Discuss the generalisability (external validity) of the study results	14
**Other information**			
Funding	22	Give the source of funding and the role of the funders for the present study and, if applicable, for the original study on which the present article is based	18

*Give information separately for exposed and unexposed groups.

Note: An Explanation and Elaboration article discusses each checklist item and gives methodological background and published examples of transparent reporting. The STROBE checklist is best used in conjunction with this article (freely available on the Web sites of PLoS Medicine at http://www.plosmedicine.org/, Annals of Internal Medicine at http://www.annals.org/, and Epidemiology at http://www.epidem.com/). Information on the STROBE Initiative is available at www.strobe-statement.org.

For peer review only
